# Alcohol drinking and risks of total and site‐specific cancers in China: A 10‐year prospective study of 0.5 million adults

**DOI:** 10.1002/ijc.33538

**Published:** 2021-03-09

**Authors:** Pek Kei Im, Iona Y. Millwood, Christiana Kartsonaki, Yiping Chen, Yu Guo, Huaidong Du, Zheng Bian, Jian Lan, Shixian Feng, Canqing Yu, Jun Lv, Robin G. Walters, Liming Li, Ling Yang, Zhengming Chen, The China Kadoorie Biobank (CKB) Collaborative Group

**Affiliations:** ^1^ Clinical Trial Service Unit and Epidemiological Studies Unit (CTSU), Nuffield Department of Population Health University of Oxford Oxford UK; ^2^ Medical Research Council Population Health Research Unit (MRC PHRU), Nuffield Department of Population Health University of Oxford Oxford UK; ^3^ Chinese Academy of Medical Sciences Beijing China; ^4^ NCDs Prevention and Control Department Liuzhou CDC Liuzhou China; ^5^ NCDs Prevention and Control Department Henan CDC Zhengzhou China; ^6^ Department of Epidemiology and Biostatistics School of Public Health, Peking University Beijing China

**Keywords:** alcohol, cancer, China, cohort studies, drinking patterns

## Abstract

Alcohol drinking is associated with increased risks of several site‐specific cancers, but its role in many other cancers remains inconclusive. Evidence is more limited from China, where cancer rates, drinking patterns and alcohol tolerability differ importantly from Western populations. The prospective China Kadoorie Biobank recruited >512 000 adults aged 30 to 79 years from 10 diverse areas during 2004 to 2008, recording alcohol consumption patterns by a standardised questionnaire. Self‐reported alcohol consumption was estimated as grams of pure alcohol per week based on beverage type, amount consumed per occasion and drinking frequency. After 10 years of follow‐up, 26 961 individuals developed cancer. Cox regression was used to estimate adjusted hazard ratios (HRs) and 95% confidence intervals (CIs) relating alcohol consumption to incidence of site‐specific cancers. Overall, 33% (n = 69 734) of men drank alcohol regularly (ie, ≥weekly) at baseline. Among male current regular drinkers, alcohol intake showed positive dose‐response associations with risks of cancers in the oesophagus (655 events; HR = 1.98 [95%CI 1.79‐2.18], per 280 g/wk), mouth and throat (236; 1.74 [1.48‐2.05]), liver (573; 1.52 [1.31‐1.76]), colon‐rectum (575; 1.19 [1.00‐1.43]), gallbladder (107; 1.60 [1.16‐2.22]) and lung (1017; 1.25 [1.10‐1.42]), similarly among never‐ and ever‐regular smokers. After adjustment for total alcohol intake, there were greater risks of oesophageal cancer in daily drinkers than nondaily drinkers and of liver cancer when drinking without meals. The risks of oesophageal cancer and lung cancer were greater in men reporting flushing after drinking than not. In this male population, alcohol drinking accounted for 7% of cancer cases. Among women, only 2% drank regularly, with no clear associations between alcohol consumption and cancer risk. Among Chinese men, alcohol drinking is associated with increased risks of cancer at multiple sites, with certain drinking patterns (eg, daily, drinking without meals) and low alcohol tolerance further exacerbating the risks.

## Abstract 2

What's new?

A comprehensive assessment of the role of alcohol in cancer aetiology is needed in China, where cancer rates, drinking patterns, and alcohol tolerability differ from those in the West. In this large prospective study, regular alcohol drinkers had increased risks of cancers in several sites previously considered to be alcohol‐related (i.e., oesophagus, mouth and throat, liver and colon‐rectum) as well as in the lung and gallbladder. Certain drinking patterns (e.g., drinking daily or without meals) and low alcohol tolerance further exacerbated the risks. The findings suggest that lowering population‐levels of alcohol consumption is an important strategy for cancer prevention.

**Abbreviations**
ALDH2: aldehyde dehydrogenase 2
BMI: body mass index
CI: confidence interval
CKB: China Kadoorie Biobank
HBsAg: hepatitis B surface antigen
HED: heavy episodic drinking
HR: hazard ratio
IARC: International Agency for Research on Cancer
ICD‐10: International Classification of Diseases, 10th Revision

## 1 INTRODUCTION

Alcohol consumption is responsible for an estimated 3 million annual deaths globally, with 75% occurring in men.^1^ In 2016, cancer accounted for 20% of alcohol‐attributable deaths among men and 27% among women aged 50+ years.^2^ In China, the burden of cancer is increasing due partly to an ageing population, and partly to changes in lifestyles, including increased alcohol consumption among men since the 2000s.^3^, ^4^

The International Agency for Research on Cancer (IARC) has concluded, based mainly on evidence from Western populations, that alcohol consumption is causally related to cancers of the oral cavity, pharynx, larynx, oesophagus, liver, colon and rectum, and female breast.^5^ However, evidence remains inconclusive for many other cancers such as lung, stomach and pancreas.^5^, ^6^, ^7^ Moreover, the relevance of different drinking patterns (eg, drinking daily, drinking with/without meals) for the risk has not been extensively investigated.^8^, ^9^ In China, previous prospective studies on alcohol and cancer were constrained by small sample size, single study area, covering a period in the distant past or lack of detailed information on drinking patterns.^10^, ^11^, ^12^, ^13^, ^14^, ^15^ More recently, the China Kadoorie Biobank (CKB) cohort of 0.5 million participants has provided new evidence about the hazards of alcohol drinking for pancreatic cancer and oesophageal cancer,^16^, ^17^ but was yet to investigate more comprehensively other cancer sites and the relevance of drinking patterns and alcohol tolerability for cancer risk. In East Asia, many people cannot metabolise alcohol effectively due to an inherited deficiency in the enzyme aldehyde dehydrogenase 2 (ALDH2), which leads to acetaldehyde accumulation in the circulation and the flushing response after moderate drinking, and may alter susceptibility to the carcinogenic effects of alcohol.^17^ Thus, comprehensive assessment of the role of alcohol in cancer aetiology is needed in China where drinking patterns (eg, drinking spirits, drinking with meals) and cancer rates (eg, high incidence of liver, stomach and oesophageal cancers) differ importantly from the West.^3^, ^18^

Using data from the CKB, this study aims to (a) assess the associations of alcohol consumption and drinking patterns with incidence of cancer, both overall and at specific sites; and (b) examine the associations of alcohol with cancer risk in certain population subgroups and by the flushing response.

## 2 MATERIALS AND METHODS

### 2.1 Study population

Details of the CKB study design and methods have been previously reported.^19^ Briefly, 512 715 adults aged 30 to 79 years were recruited from 10 areas across China during 2004 to 2008. Trained health workers administered a laptop‐based questionnaire recording sociodemographic factors, alcohol drinking, smoking, diet, physical activity and medical history; undertook physical measurements (eg, blood pressure, anthropometry); and collected a blood sample for long‐term storage and onsite hepatitis B surface antigen (HBsAg) testing (ACON Biotech). Two resurveys of ~5% randomly selected surviving participants were conducted using similar procedures in 2008 and 2013 to 2014. Ethical approval was obtained from local, national and international ethical committees. All participants provided written informed consent.

### 2.2 Assessment of alcohol drinking

Detailed questionnaire assessment of alcohol consumption has been described previously.^4^, ^18^, ^20^ Based on their past and current drinking history, participants were classified into: abstainers (had never drunk alcohol in the past year and had not drunk in most weeks in the past); ex‐regular drinkers (had not drunk alcohol in most weeks in the past year but had done so in the past); occasional drinkers (had drunk alcohol but less than weekly in the past year and had not drunk alcohol in most weeks in the past); and current regular drinkers (had drunk alcohol in most weeks in the past year). Current regular drinkers were asked further questions about their drinking patterns including drinking frequency; beverage types and amount consumed for each type on a typical drinking day; time of drinking in relation to meals; and age started drinking regularly. For this report, heavy episodic drinking (HED) was defined as consuming >60 g of alcohol on a typical drinking occasion for men and >40 g/occasion for women.^21^ The flushing response was defined by the self‐reported experience of hot flushes soon after drinking the first mouthful or a small amount of alcohol. Further details of alcohol assessment are in Table S1.

### 2.3 Follow‐up for incident cancer

The vital status of participants was obtained periodically from local death registries, supplemented by annual active confirmation through local residential, health insurance and administrative records. Cancer incident events were collected through linkage with cancer registries and the National Health Insurance databases. All events were coded with International Classification of Diseases, 10th Revision (ICD‐10), blinded to the baseline information. The list of incident site‐specific cancer endpoints examined is in Table S2. IARC alcohol‐related cancers were defined as cancers with convincing causal relevance with alcohol, as concluded by IARC,^5^ which include mouth and throat (ICD‐10: C00‐C14, C32); oesophagus (C15); colon‐rectum (C18‐C20); liver (C22); and female breast (C50). Other cancers, apart from ill‐defined neoplasms, were classified as “other cancers of known sites.” By 1 January 2017, 44 037 (8.6%) participants had died and 4781 (0.9%) were lost to follow‐up.

### 2.4 Exclusion criteria

To minimise reverse causality bias, the present study excluded participants with a previous history of cancer reported at baseline (n = 2578) from all analyses, leaving 510 137 participants (209 237 men, 300 900 women) in the study cohort. For certain specific analyses, additional exclusions were also applied, including those with prior liver cirrhosis or hepatitis (n = 6139) from analyses of liver cancer, and those with prior chronic respiratory disease (n = 43 195) from analyses of lung cancer.

### 2.5 Statistical analysis

Given that few women drank alcohol regularly, the main analyses were focused on men. Means and percentages of baseline characteristics were adjusted for age and study areas by direct standardisation. Cox regression models were used to estimate hazard ratios (HRs) for incident cancers associated with alcohol drinking status in all participants, and with alcohol consumption level and patterns among current regular drinkers, stratified by age at risk and study area, and adjusted for education, income, smoking, physical activity, fruit intake, body mass index (BMI) and family history of cancer. Analyses of drinking patterns were additionally adjusted for total weekly consumption. Comparisons of HRs of the first 5 and subsequent years of follow‐up suggested no clear evidence of violation of the proportional hazard assumption. For analyses involving comparisons of just two groups (ie, an exposure category with the reference group), conventional 95% confidence intervals (CIs) were reported. For analyses involving more than two categories of exposure, floating SEs were used to estimate group‐specific 95% CIs of the log HRs of all categories including the reference group, enabling comparison between any two categories (instead of just pairwise comparisons with the reference category).^22^

Repeat alcohol measures for participants who attended both subsequent resurveys were used to correct for regression dilution bias.^23^ To assess the shapes of the associations between usual alcohol intake and cancers, the HRs of predefined baseline consumption categories^18^ (<140, 140‐279, 280‐419, 420+ g/wk in men; <70, 70‐139, 140+ g/wk in women) were plotted against the corresponding mean usual alcohol intake, which was the average intake of the two resurveys. The associations were further examined separately in never‐regular smokers (ie, never [smoked <100 cigarettes in lifetime] or occasional [ever smoked occasionally but had never smoked regularly, that is, on most days, in lifetime] smokers) and in ever‐regular smokers (ie, ex‐regular or current regular smokers). Smoking data have been previously validated against exhaled carbon monoxide (see Supplementary Methods).^24^ Log HR estimates and corresponding SEs for baseline alcohol intake, modelled as a continuous variable, were divided by the regression dilution ratio (0.54 for men, 0.56 for women, see Table S3) to obtain estimated HRs per 280 g/wk (ie, around four drinks per day) higher usual alcohol intake. Assuming a linear association, HRs per 280 g/wk higher usual alcohol intake were compared across strata of selected baseline factors (eg, smoking, BMI, HBsAg seropositivity, flushing response), with heterogeneity in effect sizes assessed by chi‐squared tests. Departure from linearity was assessed using restricted cubic splines with five knots at the 5th, 27.5th, 50th, 72.5th and 95th percentiles of the total distribution of alcohol intake.

The population attributable fraction of cancer from ever‐regular drinking (including both ex‐regular and current‐regular drinking) was calculated as P(HR‐1)/HR, where P was the prevalence of ever‐regular drinking among those who developed the relevant cancer in CKB.^25^

Various sensitivity analyses were undertaken including (a) repeated analyses with inclusion of abstainers, occasional drinkers and ex‐regular drinkers; (b) restricting analysis to cancer mortality; (c) further adjustment for potential confounders (self‐reported health status [four categories], intake of meat and preserved vegetables [five categories]); and (d) excluding participants with self‐reported poor health or other prior chronic disease, or the first 3 years of follow‐up (see Supplementary Methods). All *P* values were two‐sided and *P* < .05 denotes statistical significance. All analyses used SAS (version 9.4) and R (version 3.6.1).

## 3 RESULTS

Among the 510 137 participants, the mean age was 52 (SD 10.7) years, 41% were men and 56% were rural residents. Among men, 33% (n = 69 734) reported drinking alcohol at least weekly (Table 1), compared to 2% of women (n = 6219) (Table S4). Among men, abstainers and ex‐regular drinkers tended to be older and rural residents, and have prior chronic disease than occasional and current regular drinkers. Compared with moderate drinkers (ie, <140 g/wk), heavier drinkers tended to be rural residents and less educated, smoke regularly, consume less fresh fruit and have higher mean of blood pressure. Male current regular drinkers consumed on average 286 g of alcohol per week, with 18% reporting the flushing response, 37% engaging in HED, 62% drinking daily, 70% drinking spirits and 86% drinking with meals (Table S5). Female current regular drinkers had lower consumption (mean 116 g/wk) than men (Table S6).

**TABLE 1:** Baseline characteristics of participants by alcohol drinking categories in men

					Current regular drinkers				
	Overall	Abstainers	Ex‐regular drinkers	Occasional drinkers	All current regular	<140 g/wk	140–279 g/wk	280–419 g/wk	420+ g/wk
Number of participants	209 237	42 479	18 061	78 963	69 734	24 999	18 874	12 811	13 050
Sociodemographic characteristics									
Mean age, y (SD)	52.8 (10.9)	56.3 (11.1)	56.9 (10.3)	51.2 (10.8)	51.5 (10.2)	51.7 (11.1)	51.9 (10.2)	51.2 (9.6)	51.4 (9.5)
Urban area, %	43.4	31.1	41.0	44.1	49.9	58.8	52.3	47.9	30.8
Educational attainment >6 y, %	57.8	54.5	56.7	60.5	57.7	60.7	57.1	57.9	53.9
Income >20 000 yuan/y, %	45.7	42.0	45.0	46.7	46.8	47.8	46.3	45.4	46.8
Married, %	92.9	91.4	93.4	93.3	93.3	94.0	93.3	93.3	92.1
Lifestyle factors									
Regular smoking, %	61.2	52.5	60.8	56.9	71.8	65.8	73.1	76.4	79.9
Daily fresh fruit consumption, %	23.0	24.9	25.3	25.2	21.1	25.2	20.3	17.8	15.8
Physical activity, mean MET‐h/d (SD)	22.1 (15.3)	21.2 (15.1)	20.3 (14.5)	22.6 (15.6)	22.4 (15.0)	22.7 (15.1)	23.1 (14.9)	23.3 (15.4)	22.8 (15.2)
Daily tea drinking, %	40.9	36.1	40.3	37.3	48.5	45.8	47.8	49.0	52.5
Physical measurements, mean (SD)									
Body mass index, kg/m^2^	23.4 (3.2)	23.3 (3.2)	23.9 (3.4)	23.4 (3.2)	23.4 (3.2)	23.7 (3.2)	23.7 (3.2)	23.7 (3.2)	23.7 (3.2)
Systolic blood pressure, mmHg	132.8 (20.0)	132.4 (21.5)	134.4 (21.5)	131.0 (18.8)	134.8 (19.8)	131.6 (21.5)	134.3 (19.8)	136.0 (19.9)	137.4 (20.7)
Diastolic blood pressure, mmHg	79.2 (11.4)	78.6 (11.5)	80.0 (11.7)	78.1 (10.9)	80.6 (11.5)	79.5 (11.5)	81.0 (11.5)	82.2 (11.6)	83.0 (11.7)
Health and medical history, %^a^									
Poor health	8.8	12.5	16.9	7.6	5.8	6.0	5.9	5.4	6.6
Any chronic disease^b^	22.2	26.6	37.1	20.9	17.7	18.9	17.6	17.1	17.7
Coronary heart disease	2.7	3.3	5.2	2.3	2.0	2.2	1.9	1.7	2.3
Stroke/transient ischaemic attack	2.3	3.6	6.0	1.6	1.3	1.4	1.3	1.1	1.3
Liver cirrhosis/chronic hepatitis	1.7	2.7	3.8	1.6	1.2	1.1	1.2	1.2	1.5
Emphysema/bronchitis	3.1	3.9	4.7	2.7	2.5	2.5	2.6	2.2	2.7
Chronic obstructive pulmonary disease	8.8	10.4	10.8	7.7	8.3	8.0	8.5	7.7	9.7
Diabetes	5.5	6.6	8.8	5.0	4.6	4.6	4.4	5.0	5.6
Family history of cancer	16.5	14.6	18.0	16.5	17.2	16.9	17.4	17.6	17.7

*Note:* Participants with self‐reported prior cancer were excluded. Prevalences and means are adjusted for age and study areas as appropriate.

Abbreviation: MET‐h/d, metabolic equivalents of task per hours per day.

^a^ All self‐reported except for chronic obstructive pulmonary disease and diabetes, which included both self‐reported and screen‐detected events.

^b^ Chronic diseases included self‐reported coronary heart disease, stroke, transient ischaemic attack, diabetes, tuberculosis, chronic hepatitis/liver cirrhosis, rheumatoid arthritis, peptic ulcer, emphysema/bronchitis, gallstone/gallbladder disease and kidney disease.

### 3.1 Alcohol drinking status and cancer risk

During 5 million person‐years of follow‐up (median 10 years), 26 961 individuals (13 342 men, 13 619 women) developed cancer. Among men, the risks of total and most site‐specific cancers tended to be higher among current and ex‐regular drinkers, and lower among occasional drinkers, than abstainers (Table 2). Compared with abstainers, current regular drinkers had 26% (95% CI 16%‐36%) and 7% (2%‐12%) higher risks for IARC alcohol‐related cancers and for total cancer, respectively. Among women, there were no clear associations of alcohol drinking with IARC alcohol‐related cancers or with other cancers (Tables S7 and S8, Figure S1), but the numbers of cases among regular drinkers were extremely small.

**TABLE 2:** Adjusted hazard ratios (HRs) for incident cancers associated with alcohol drinking status in men

		Abstainers		Ex‐regular drinkers		Occasional drinkers		Current regular drinkers				
Cancer site	All men N	N	HR (95% CI)	N	HR (95% CI)	N	HR (95% CI)	N	HR (95% CI)	*P* ^a^	HR (95% CI) per 280 g/wk	*P*_trend_ ^b^
Mouth and throat	541	90	1.00 (0.81‐1.24)	61	1.46 (1.13‐1.88)	154	1.22 (1.03‐1.44)	236	1.73 (1.51‐1.99)	<.001	1.74 (1.48‐2.05)	<.001
Oesophagus	1608	243	1.00 (0.88‐1.14)	152	1.23 (1.05‐1.44)	558	1.05 (0.96‐1.15)	655	1.80 (1.66‐1.96)	<.001	1.98 (1.79‐2.18)^c^	<.001
Colon and rectum	1527	306	1.00 (0.89‐1.13)	203	1.27 (1.10‐1.46)	443	0.95 (0.86‐1.05)	575	1.20 (1.10‐1.31)	.02	1.19 (1.00–1.43)	.051
Colon	856	180	1.00 (0.86‐1.17)	118	1.28 (1.06‐1.53)	255	0.93 (0.81‐1.05)	303	1.11 (0.98‐1.25)	.31	1.13 (0.87–1.45)	.36
Rectum	946	185	1.00 (0.86‐1.16)	122	1.22 (1.02‐1.46)	261	0.93 (0.82‐1.06)	378	1.26 (1.13‐1.40)	.02	1.29 (1.04–1.58)	.02
Liver	1651	378	1.00 (0.90‐1.11)	208	1.23 (1.07‐1.41)	492	0.83 (0.76‐0.92)	573	1.06 (0.97‐1.16)	.39	1.52 (1.31–1.76)	<.001
Stomach	2221	520	1.00 (0.91‐1.10)	242	1.00 (0.88‐1.13)	702	0.87 (0.81‐0.95)	757	1.02 (0.94‐1.10)	.77	1.11 (0.94‐1.30)	.22
Pancreas	405	89	1.00 (0.80‐1.25)	40	0.96 (0.70‐1.32)	111	0.97 (0.80‐1.18)	165	1.26 (1.07‐1.49)	.10	1.20 (0.86‐1.66)	.28
Lung	2741	660	1.00 (0.92‐1.08)	344	1.07 (0.97‐1.20)	720	0.80 (0.74‐0.86)	1017	0.96 (0.90‐1.03)	.48	1.25 (1.10–1.42)	<.001
Gallbladder and biliary tract	279	54	1.00 (0.76‐1.32)	29	1.02 (0.71‐1.48)	89	1.31 (1.05‐1.64)	107	1.33 (1.09‐1.62)	.11	1.60 (1.16–2.22)	.004
Skin	137	41	1.00 (0.72‐1.39)	17	1.00 (0.61‐1.62)	44	0.83 (0.61‐1.14)	35	0.65 (0.46‐0.92)	.08	0.80 (0.36‐1.81)	.60
Prostate	402	110	1.00 (0.82‐1.22)	64	1.25 (0.97‐1.60)	120	1.00 (0.83‐1.21)	108	0.89 (0.73‐1.08)	.41	1.17 (0.77‐1.77)	.47
Kidney	219	34	1.00 (0.70‐1.43)	28	1.52 (1.04‐2.21)	73	1.15 (0.91‐1.47)	84	1.27 (1.01‐1.60)	.26	1.42 (0.90‐2.23)	.13
Bladder	356	89	1.00 (0.80–1.25)	57	1.14 (0.88‐1.49)	96	0.73 (0.60‐0.91)	114	0.75 (0.61‐0.90)	.053	0.84 (0.52‐1.34)	.46
Brain	227	48	1.00 (0.74‐1.35)	29	1.12 (0.77‐1.62)	80	0.92 (0.73‐1.16)	70	0.82 (0.64‐1.04)	.31	1.35 (0.89‐2.07)^c^	.16
Thyroid	84	12	1.00 (0.55‐1.82)	10	1.66 (0.88‐3.13)	27	0.96 (0.65‐1.43)	35	1.18 (0.83‐1.68)	.63	1.10 (0.55‐2.22)	.78
Lymphoma	402	104	1.00 (0.82‐1.23)	49	1.02 (0.77‐1.36)	115	0.88 (0.73‐1.07)	134	0.92 (0.77‐1.10)	.56	1.24 (0.91‐1.69)	.18
Multiple myeloma	137	34	1.00 (0.70‐1.43)	10	0.62 (0.33‐1.16)	44	0.89 (0.65‐1.21)	49	1.01 (0.75‐1.36)	.96	1.46 (0.86‐2.51)	.16
Leukaemia	287	63	1.00 (0.77‐1.30)	31	1.15 (0.80‐1.64)	86	0.87 (0.70‐1.09)	107	1.14 (0.94‐1.40)	.43	1.52 (1.08‐2.12)	.01
Other less common cancers of known sites	935	208	1.00 (0.87‐1.15)	122	1.21 (1.01‐1.45)	280	0.94 (0.83‐1.06)	325	1.05 (0.94‐1.18)	.60	1.08 (0.85‐1.37)	.54
IARC alcohol‐related cancers	5403	1056	1.00 (0.94‐1.07)	673	1.30 (1.20‐1.40)	1669	0.92 (0.88‐0.97)	2005	1.26 (1.20‐1.32)	<.001	1.65 (1.53–1.77)^c^	<.001
Other cancers of known sites (non‐IARC alcohol‐related)	8730	2085	1.00 (0.96‐1.05)	1076	1.07 (1.00‐1.13)	2523	0.87 (0.83‐0.90)	3046	0.99 (0.95‐1.03)	.76	1.17 (1.09–1.27)^c^	<.001
All cancers^d^	13 342	2991	1.00 (0.96‐1.04)	1647	1.14 (1.08‐1.20)	3947	0.88 (0.85‐0.91)	4757	1.07 (1.04‐1.10)	.006	1.37 (1.30‐1.45)^c^	<.001

*Note:* Cox models are stratified by age at risk and study area, and adjusted for education, income, smoking, physical activity, fruit intake, body mass index and family history of cancer. Participants with self‐reported prior cancer were excluded from all analyses. Participants with self‐reported prior chronic hepatitis/liver cirrhosis were further excluded from analysis of liver cancer, and participants with self‐reported prior tuberculosis, emphysema/bronchitis or chronic obstructive pulmonary disease were further excluded from analysis of lung cancer.

Abbreviations: CI, confidence interval; HR, hazard ratio; IARC, International Agency for Research on Cancer.

^a^ *P* value for association comparing current regular drinkers vs abstainers.

^b^ *P* value for alcohol consumption (g/wk) modelled as a continuous variable among current regular drinkers.

^c^ The association with alcohol intake among current regular drinkers appeared nonlinear for oesophageal cancer (*P* < .0001), brain cancer (*P* = .027), IARC alcohol‐related cancer (*P* = .003), other cancers of known sites (*P* = .002) and total cancer (*P* = .006).

^d^ All cancers included ill‐defined neoplasm and are patient‐based.

### 3.2 Amount of alcohol consumption and cancer risk

**FIGURE 1:**
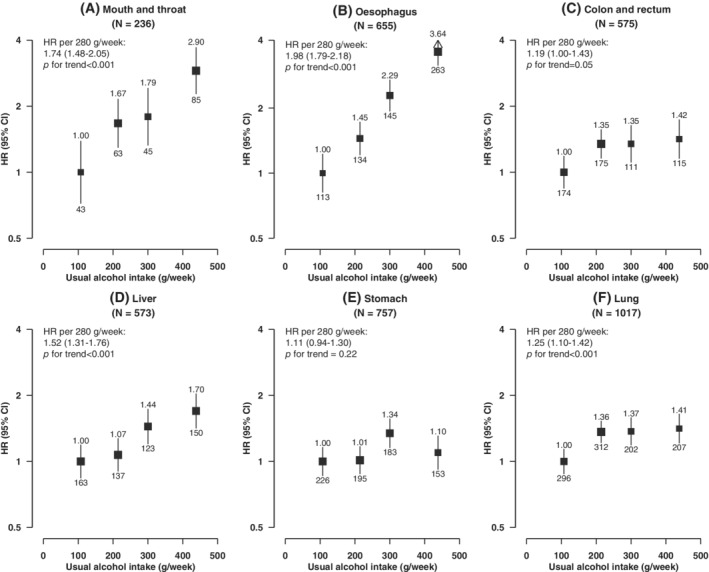
Associations of alcohol consumption with common cancers in male current regular drinkers. Cox models are stratified by age at risk and study area, and adjusted for education, income, smoking status, physical activity, fresh fruit intake, body mass index and family history of cancer. A‐D, Classified as IARC alcohol‐related cancers. Each solid square represents HR with the area inversely proportional to the “floated” variance of the log HR. The vertical lines indicate group‐specific 95% CIs. The numbers above the error bars are the point estimates for HRs, and the numbers below are the number of events. Alcohol intake is classified based on baseline consumption of <140, 140 to 279, 280 to 419 and ≥420 g/wk. *P* for trend is estimated by modelling alcohol consumption (g/wk) as a continuous variable among current regular drinkers. CI, confidence interval; HR, hazard ratio; IARC, International Agency for Research on Cancer

Among male current regular drinkers, alcohol intake was positively associated with risks of several IARC alcohol‐related cancers (Figure 1A‐D). After adjusting for regression dilution bias, each 280 g/wk higher usual alcohol intake was associated with HRs of 1.98 (95% CI 1.79‐2.18) for cancers in the oesophagus, 1.74 (1.48‐2.05) for mouth and throat, 1.52 (1.31‐1.76) for liver and 1.19 (1.00‐1.43) for colon‐rectum, with a slightly higher, although nonsignificant, HR for rectal cancer (1.29 [1.04‐1.58]) than for colon cancer (1.13 [0.87‐1.45]) (Figure S2). Within the mouth and throat cancer category, there were clear dose‐response associations of alcohol drinking with lip and oral cavity cancer, pharyngeal cancer and laryngeal cancer when examined separately (Table S9, Figure S3).

Alcohol intake was also significantly positively associated with the risks of lung cancer (1.25 [1.10‐1.42] per 280 g/wk, and *P*_trend_ < .001) (Figure 1F), with no evidence of heterogeneity between never‐regular smokers and ever‐regular smokers (*P*_heterogeneity_ = .58) (Figure S4), and of gallbladder cancer (1.60 [1.16‐2.22] per 280 g/wk) (Figure S5). The risk of brain cancer tended to increase with alcohol intake, but the number of events (n = 70) was small (Figure S6). There were no clear associations with stomach cancer (Figure 1E) or with other less common cancers (Figures S5 and S6).

**FIGURE 2:**
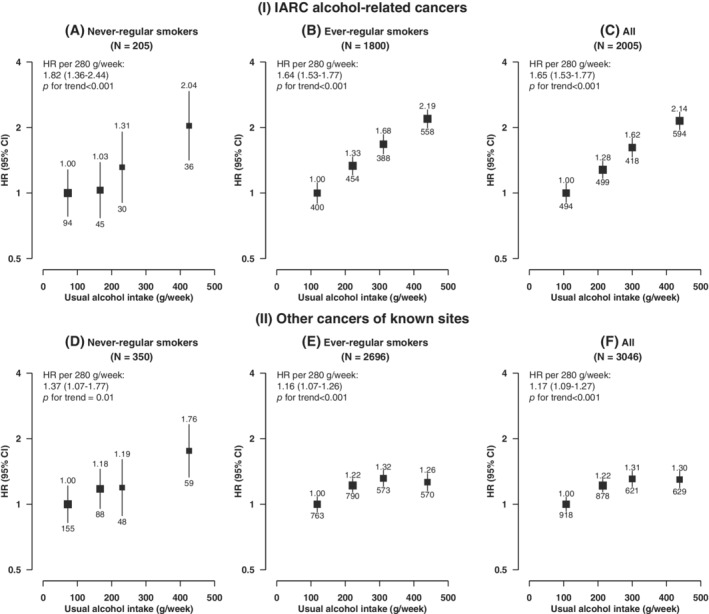
Associations of alcohol consumption with IARC alcohol‐related cancers and other cancers by smoking status in male current regular drinkers. Conventions are as in Figure 1. IARC, International Agency for Research on Cancer

Overall, the HRs per 280 g/wk higher usual alcohol intake were 1.65 (1.53‐1.77) for IARC alcohol‐related cancers, 1.17 (1.09‐1.27) for other cancers of known sites and 1.37 (1.30‐1.45) for total cancer, with similar dose‐response associations when stratified by smoking status (Figure 2, Figure S7). There was evidence of nonlinear associations for oesophageal cancer, IARC alcohol‐related cancers, other cancers of known sites and total cancer (*P*_nonlinearity_ < .007), and for brain cancer (*P*_nonlinearity_ = .03), which appeared to be largely due to a slight flattening of the splines in the small subset of extremely heavy drinkers (Figure S8).

Among men, the associations of alcohol intake with IARC alcohol‐related cancers and with total cancer were similar across subgroups defined by age, study area, education, income, BMI, physical activity and fresh fruit intake (Figure S9). The associations for liver cancer and for other common cancers were similar by HBsAg sero‐status (Figure S10).

### 3.3 Drinking patterns and flushing response with cancer risk

**FIGURE 3:**
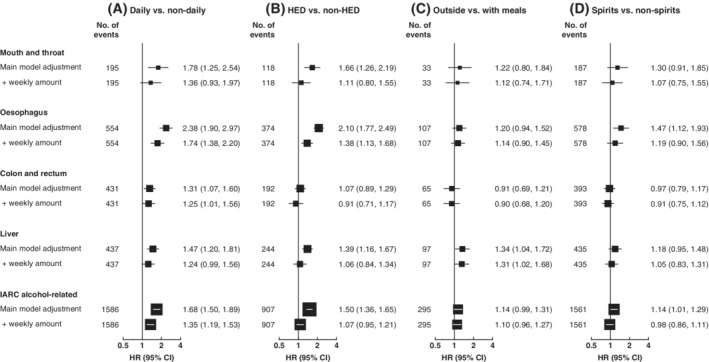
Adjusted HRs for IARC alcohol‐related cancers associated with drinking patterns in male current regular drinkers. Cox models are stratified by age at risk and study area, and adjusted for education, income, smoking status, physical activity, fresh fruit intake, body mass index and family history of cancer, and total weekly intake where indicated. Heavy episodic drinking (HED) is defined as drinking >60 g/session. Conventions are as in Figure 1. CI, confidence interval; HR, hazard ratio; IARC, International Agency for Research on Cancer

**FIGURE 4:**
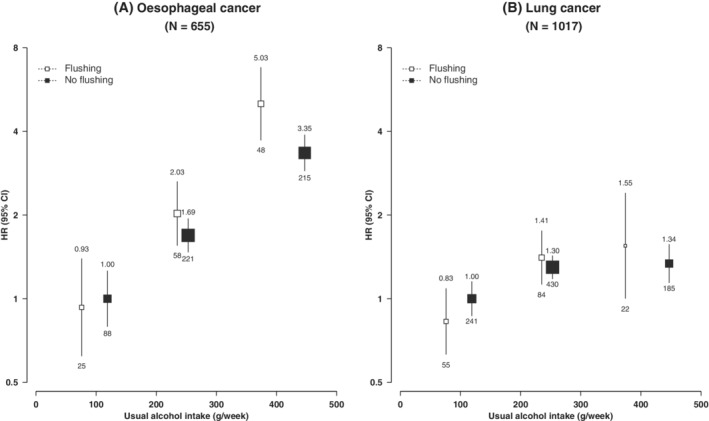
Joint associations of alcohol consumption and flushing status with oesophageal cancer and lung cancer in male current regular drinkers. Alcohol intake, separately in men reporting flushing and in others reporting no flushing, is classified based on baseline consumption of <140, 140 to 419 and ≥420 g/wk. Conventions are as in Figure 1

In men, daily drinking and HED were associated with increased risks of most specific IARC alcohol‐related cancers, while drinking without meals was associated with increased liver cancer risk and drinking spirits was associated with increased oesophageal cancer risk (Figure 3). After further adjusting for total alcohol intake, most of these associations attenuated to the null; however, the excess risks of oesophageal cancer and colorectal cancer associated with daily drinking persisted, as did the excess oesophageal cancer risk with HED and the excess liver cancer risk with drinking without meals. Across strata of weekly consumption, the excess risks of total cancer and IARC alcohol‐related cancers, especially oesophageal cancer, associated with daily drinking remained significant among those drinking <280 g/wk, while the HED‐associated excess risks became nonsignificant (Tables S10 and S11). Given amount consumed, the risks of IARC alcohol‐related cancers and other cancers increased with duration of regular drinking (Figure S11). The associations of alcohol intake tended to be stronger among those reporting flushing after drinking than those not reporting flushing for certain cancers, particularly oesophageal cancer and lung cancer (Figure S12). However, the difference in the dose‐response relationships by flushing status was less clear for lung cancer (Figure 4).

### 3.4 Sensitivity analyses

The dose‐response associations among current regular drinkers were similar with further adjustment for poor self‐reported health and meat and preserved vegetable intake (Table S12). Inclusion of abstainers, occasional drinkers and ex‐regular drinkers in the analyses showed an overall J‐shaped association of alcohol drinking with total cancer and most major cancer sites (Figures S13‐S16). Further excluding the first 3 years of follow‐up or participants with poor health or prior chronic disease increased risk estimates comparing current regular drinkers with abstainers, particularly for IARC alcohol‐related cancers, but did not alter the dose‐response associations among current regular drinkers (Tables S13 and S14).

If the observed associations between alcohol drinking and cancers are largely causal, in this study population ever‐regular alcohol drinking accounted for 7.2% of total cancer cases among men, including 12.4% of IARC alcohol‐related cancers and almost 20% of upper aero‐digestive tract cancers (Table S15).

## 4 DISCUSSION

In this large prospective study in China, one third of men reported drinking alcohol regularly and among them there were significantly increased risks of cancers in several sites previously considered to be alcohol‐related (ie, oesophagus, mouth and throat, liver, colon‐rectum) and also in certain other sites (eg, lung, gallbladder). For most of these cancers, there were clear dose‐response relationships with the amount consumed, and the associations persisted when restricting analyses to never‐regular smokers or excluding early follow‐up. For oesophageal cancer and lung cancer, the risks appeared greater among men reporting flushing after drinking. Moreover, given amount consumed, those who drank daily had elevated cancer risks compared with nondaily drinkers, particularly for oesophageal cancer. As very few women in the study drank alcohol regularly, the associations with cancer in women could not be evaluated effectively.

Many previous studies have reported a J‐shaped relationship, which was also observed in the present study, between alcohol and total cancer risk when considering the whole study population (ie, drinkers and nondrinkers at the same time).^8^, ^12^ However, the shape of the association may be affected unduly by reverse causation (eg, sick quitters) and potentially residual confounding (systematic differences between drinkers and nondrinkers, which are hard to measure and may affect cancer risk, eg, long‐standing illness and social disadvantages), hence underestimating the hazards of alcohol drinking.^26^, ^27^, ^28^ In our study, we restricted the main analyses to current regular drinkers in order to reliably assess the dose‐response relationships between amount of alcohol intake and cancer risk, and found a steeper dose‐response relationship in men than previous Chinese studies.^13^, ^14^ Our alcohol‐attributable cancer burden estimate (7.2%) was somewhat higher than previous estimations for China (5.9%)^29^ and for the world (5.8%),^1^ possibly reflecting the differences in relative risk estimates (derived from Western vs Chinese population), study design (case‐control vs cohort), rates of different cancer sites and drinking prevalence between other studies and our study.

Studies in different populations have consistently shown strong positive associations between alcohol consumption and cancers of the mouth and throat and the oesophagus, particularly oesophageal squamous cell carcinoma, which is highly prevalent in China.^5^, ^6^, ^7^, ^30^ In China, while previous evidence on alcohol consumption and oesophageal cancer was robust, the evidence on mouth and throat cancer was limited.^10^, ^12^, ^14^ In CKB, we demonstrated clear associations of alcohol intake not only with oesophageal cancer but also with mouth and throat cancer, both of which were the strongest among all site‐specific cancers observed. Our risk estimates were broadly similar to those by the World Cancer Research Fund (WCRF) (19% vs 25% higher risk per 10 g/d for oesophagus, 15% vs 9%‐19% for mouth and throat, CKB vs WCRF).^6^

Previous studies in Western and high‐income East Asian populations have reported dose‐response associations between alcohol intake and liver cancer.^5^, ^6^ However, evidence was limited in China that accounts for half of global liver cancer cases and deaths, predominantly attributed to chronic hepatitis B virus (HBV) infection.^31^, ^32^ In China, a meta‐analysis of 18 case‐control studies reported an excess liver cancer risk in drinkers vs nondrinkers (odds ratio 1.56, 3800 cases),^33^ but prospective studies found no significant associations with drinking status or amount consumed.^12^, ^13^, ^14^ These previous cohort studies were mostly conducted in the 1980s to 1990s, when other major liver cancer risk factors (eg, HBV infection, aflatoxin) were more prevalent in China and alcohol consumption level was lower.^3^ In the CKB, we found significant dose‐response associations of alcohol intake with liver cancer incidence, consistently among individuals with different HBV infection status, and with liver cancer mortality (Tables S16 and S17), suggesting the increasing importance of heavy alcohol intake as a risk factor for liver cancer in China. Our risk estimate appeared somewhat higher than the WCRF estimate (11% vs 4% higher risk per 10 g/d),^6^ which had excluded individuals who were carriers of or infected with hepatitis.^34^ In the present study, however, there was no evidence of apparent effect modification on alcohol and cancer relationship by HBV infection, although statistical power was limited.

Increasing colorectal cancer incidence is considered a marker of socioeconomic development,^35^ and rapid increases have been seen in recent decades in China in parallel with rapid economic development and urbanisation,^3^ and adoption of western lifestyles including increased alcohol consumption.^4^ Despite a clear positive association between alcohol consumption and colorectal cancer risk reported from high‐income countries,^6^, ^36^, ^37^ inconsistent findings have been presented in previous Chinese studies mostly conducted in the 1980s to 1990s when alcohol consumption levels were lower.^12^, ^13^, ^14^, ^15^ Our study, with more incident colorectal cancer cases than all previous Chinese studies combined, showed a clear increased colorectal cancer risk associated with alcohol consumption, in a similar magnitude to that reported from high‐income populations (HR 1.04 in CKB vs 1.02‐1.08 per 10 g/d).^6^, ^37^, ^38^ Consistent with Western studies,^36^, ^37^ we also demonstrated a slightly stronger association for rectal cancer than for colon cancer, although the difference was nonsignificant.

For lung cancer, previous cohort studies tended to show J‐shaped associations with alcohol consumption when considering drinkers and nondrinkers at the same time.^6^, ^7^ There is evidence mainly from Western and Japanese populations that the associations differed by smoking status, with strong positive associations observed among current or heavier smokers^39^ but generally no significant associations among never smokers.^40^ In China, two prospective studies reported excess lung cancer mortality in heavy drinkers after adjusting for smoking, but did not investigate the associations separately by smoking status.^12^, ^14^ In CKB, we observed a significant dose‐response relationship between alcohol intake and lung cancer, among both never‐ and ever‐regular smokers, with the risk estimates stronger than that in the WCRF report (6% in CKB vs 3% higher risk per 10 g/d).^6^ Our findings among never‐regular smokers, though with limited power (71 cases), were consistent with a pooled analysis of seven Western cohorts that involved similar small number of cases (74 events).^41^ Future studies with much more cases are needed to confirm (or refute) the associations between alcohol drinking and lung cancer among never smokers. For stomach cancer, previous studies in China and other populations have reported excess risks in heavy drinkers,^5^, ^6^, ^7^, ^11^, ^14^, ^42^, ^43^, ^44^, ^45^, ^46^, ^47^ but most of these studies lacked adjustment for any dietary factors (eg, fresh fruit, red meat, preserved vegetables)^14^, ^46^, ^47^ or were based on case‐control studies.^42^, ^43^, ^45^ In CKB, no apparent dose‐response relationships of alcohol consumption with stomach cancer were observed.

Existing evidence from high‐income populations has suggested possible links between alcohol consumption and several other cancers, but relevant prospective evidence is limited in China. The positive association between alcohol intake and gallbladder cancer in CKB was broadly consistent with previous reports in non‐Chinese populations.^7^ However, despite previous reports of inverse associations with kidney cancer and non‐Hodgkin lymphoma, mostly in Western populations,^6^, ^7^ we found no similar associations in Chinese adults. Our null findings might be due to limited statistical power and the combination of Hodgkin and non‐Hodgkin lymphoma, which might have different associations with alcohol consumption.

In a few studies that have examined the relationships between drinking patterns and cancer risks, most tended to focus on aggregate outcomes only or lacked appropriate adjustment for total intake.^8^, ^9^, ^10^, ^48^ Previous studies from the United States (1167 cases) and Japan (3050 deaths) have shown that more frequent drinking conferred higher alcohol‐related cancer risk and total cancer mortality in men.^8^, ^9^ Our study is the first to systematically investigate the associations of drinking patterns with total and site‐specific cancers in a Chinese population. The persistent excess cancer risks by daily drinking, especially for oesophageal cancer, and by prolonged duration of regular drinking, given the amount consumed, suggested the detrimental effects of certain drinking patterns (eg, repeated and long‐term exposure to alcohol) on cancer risk. In China, consuming alcohol with meals is much more common than in Western populations (86% vs 50‐60%).^49^, ^50^ Consistent with Western studies on liver cirrhosis,^49^ drinking without meals was associated with excess liver cancer risk in Chinese men, which may be related to the faster absorption of alcohol into the bloodstream in the absence of food.^51^ In China, strong spirits, rather than wine and beer, are the most common beverage types.^1^ However, in our study, there were no differences between spirits and other beverage types on IARC alcohol‐related cancer risk after adjusting for total amount consumed.

The biological mechanisms underlying the associations between alcohol consumption and cancer are not fully understood. A major pathway is via the metabolism of ethanol into carcinogenic acetaldehyde, particularly in the upper and lower gastrointestinal tract.^5^ The East Asian‐specific *ALDH2*‐rs671 loss‐of‐function variant, which leads to the accumulation of acetaldehyde causing the Asian flushing response after drinking, can be used to investigate this causal pathway and to assess how the carcinogenic effects of alcohol may be modified by alcohol tolerability.^52^ Accumulating evidence using the *ALDH2*‐rs671 gene variant has shown that ALDH2‐deficiency increases the oesophageal cancer risk associated with alcohol drinking.^17^ Likewise, we observed stronger associations of alcohol intake with oesophageal cancer and also lung cancer in men with the flushing response. Further investigations of other cancer types, and using the *ALDH2*‐rs671 gene variant, are warranted. Other possible mechanisms may include the following: the induction of cytochrome P450 2E1 by chronic drinking and associated oxidative stress, particularly for liver cancer; the solvent role of alcohol for tobacco carcinogens for upper digestive and respiratory tract cancers; alcohol‐induced alterations of serum levels of hormones and related signalling pathways for breast cancer; alcohol‐related liver cirrhosis for liver cancer; and alcohol‐induced changes in folate metabolism particularly for colorectal cancer.^5^

The chief strengths of this study include the prospective design, large study population, comprehensive adjustments for potential confounders and large numbers of incident events for a wide range of cancer sites traced via comprehensive and complete follow‐up. The exclusions of prior diseases and early follow‐up reduced reverse causality. Moreover, the repeat alcohol measures enabled adjustment for regression dilution bias.^23^ However, our study has several limitations, including limited statistical power for noncommon cancer sites and analyses among women. Also, alcohol exposure was self‐reported. Nevertheless, the baseline self‐reported alcohol intake data had good reproducibility (weighted *κ* coefficient = 0.79) in a representative subset resurveyed immediately after the baseline survey (Tables S18‐S20) and was positively correlated with blood pressure and gamma‐glutamyl transferase (Table S21), as expected, and consistent with the causal associations seen with genotype‐predicted alcohol intake,^26^ suggesting good quality of the self‐reported alcohol intake data in CKB. However, measurement error is a known occurrence when alcohol intake is self‐reported, which could affect the observed associations.^53^ For example, heavy drinking may be generally underreported, which could likely lead to underestimation of the associated cancer risks. Moreover, although our separate analyses among never‐regular smokers showed similar findings, it is possible that potential residual confounding might exist in never‐regular smokers due to potential misreporting of smoking status. Nevertheless, the self‐reported smoking status has been validated using an exhaled carbon monoxide test^24^ and by its clear associations with increased tobacco‐attributed mortality and lung cancer risk.^54^ In our study, however, it was not possible to redefine smoking status according to the levels of measured exhaled carbon monoxide, for they only reflect exposure within 24 hours and are also affected by exposure to household air pollution (eg, use of solid fuels for cooking and heating), which is common in our study population. Despite this, any residual confounding due to underreported smoking among never‐regular smokers is likely to be minimal. Finally, although careful adjustments were made, residual confounding by unknown or unmeasured factors (eg, *Helicobacter pylori* infection) might remain.

Reverse causation and residual confounding are major limitations of observational studies that can be overcome with a Mendelian randomisation (MR) approach. A recent MR study in European descents has shown suggestive positive relationships between alcohol consumption and IARC alcohol‐related cancers, but the findings were nonsignificant due in part to the weak genetic instrument used for alcohol.^55^ In Chinese populations, the two common gene variants, *ALDH2*‐rs671 and *ADH1B*‐rs1229984, which jointly predict large absolute differences in alcohol intake, offer unique opportunities for MR studies to reliably assess the causal associations between alcohol consumption and various site‐specific cancers and/or other diseases, as has been shown for stroke, demonstrating that the apparent protective associations for moderate drinking were largely not causal.^26^ Nevertheless, for cancers for which acetaldehyde is a causal factor, the potential interactions between alcohol intake and *ALDH2*‐rs671 gene variant (which is associated with both lower alcohol consumption but elevated acetaldehyde level and thus potentially higher cancer risks given the same amount of alcohol consumed) should be additionally considered in future research.

In conclusion, among Chinese men, alcohol consumption was associated with increased risks of several site‐specific cancers, some known while others less clearly established to be alcohol‐related previously. In addition to total amount, certain drinking patterns (eg, drinking daily) and reduced alcohol tolerance may further exacerbate the risks for certain cancers, especially oesophageal cancer. Lowering population‐levels of alcohol consumption is an important strategy for prevention of cancer.

## CONFLICT OF INTEREST

The authors declare no conflicts of interest.

## ETHICS STATEMENT

Ethical approval was obtained from the Ethical Review Committee of the Chinese Centre for Disease Control and Prevention (Beijing, China) and the Oxford Tropical Research Ethics Committee, University of Oxford (UK), and all participants provided written informed consent.

## Supporting information

**AppendixS1**: Supporting Information

## Data Availability

The datasets used and analysed during the current study are available from the corresponding author on reasonable request.
