# Supplementary material for: Alcohol drinking and risks of total and site‐specific cancers in China: A 10‐year prospective study of 0.5 million adults
Source: Int J Cancer. 2021 Mar 9;149(3):522–34. doi: 10.1002/ijc.33538 (PMC8359462; doi:10.1002/ijc.33538)
Supplement: Supplementary file 1 — AppendixS1: Supporting Information [file IJC-149-522-s001.pdf]

Supplementary material

Alcohol drinking and risks of total and site-specific cancers in China: a 10-year prospective study of 0.5 million adults

Pek Kei Im, MPhil[1]; Iona Y Millwood, DPhil[1,2]; *Christiana* Kartsonaki, DPhil[1,2]; Yiping Chen, DPhil[1,2]; Yu Guo, MSc[3]; Huaidong Du, PhD[1,2]; Zheng Bian, MSc[3]; Jian Lan, BSc[4]; Shixian Feng, BSc[5]; Canqing Yu, MD, PhD[6]; Jun Lv, MD, PhD[6]; Robin G Walters, PhD[1,2]; Liming Li, MPH[6]; Ling Yang, PhD[1,2]; Zhengming Chen, DPhil[1,2]; on behalf of the China Kadoorie Biobank (CKB) collaborative group

Content List of Supplementary Material

Members of the China Kadoorie Biobank collaborative group 3

Supplementary Methods 4

Table S1. Definitions of main alcohol drinking patterns 6

Table S2. Number of incident cancer cases reported in men and women without prior cancer in the CKB 7

Table S3. Estimated regression dilution ratios of alcohol intake in men and women 8

Table S4. Baseline characteristics of participants by alcohol drinking categories, in women 9

Table S5. Baseline drinking characteristics of participants by level of alcohol consumption, in male current regular drinkers 10

Table S6. Baseline drinking characteristics of participants by level of alcohol drinking, in female current regular drinkers 11

Table S7. Adjusted HRs for incident cancers associated with drinking status, in women 12

Figure S1. Associations of alcohol consumption with incident cancers, in female current regular drinkers 13

Table S8. Adjusted HRs for incident cancers associated with level of alcohol consumption, in female current regular drinkers 14

Figure S2. Associations of alcohol consumption with colon cancer and rectal cancer, in male current regular drinkers 15

Table S9. Adjusted HRs for lip and oral cavity cancer, pharyngeal cancer, and laryngeal cancer associated with alcohol drinking, in men 16

Figure S3. Associations of alcohol consumption with lip and oral cavity cancer, pharyngeal cancer, and laryngeal cancer, in male current regular drinkers 17

Figure S4. Associations of alcohol consumption with lung cancer, by smoking status in male current regular drinkers 18

Figure S5. Associations of alcohol consumption with other site-specific cancers, in male current regular drinkers 19

Figure S6. Associations of alcohol with other less common site-specific cancers, in male current regular drinkers 20

Figure S7. Associations of alcohol consumption with total cancer, by smoking status in male current regular drinkers 21

Figure S8. HRs of oesophageal cancer, IARC alcohol-related cancers, other cancers, and total cancer associated with alcohol intake among male current regular drinkers over the range of 0-2000 g/week 22

Figure S9. Adjusted HRs per 280 g/week higher usual alcohol intake for IARC alcohol-related cancers and total cancer, by population subgroups in male current regular drinkers 23

Figure S10. Adjusted HRs per 280 g/week higher usual alcohol intake for common cancers, by hepatitis B infection status in male current regular drinkers 24

Table S10. Adjusted HRs for common cancers associated with daily drinking, by total weekly amount in male current regular drinkers 25

Table S11. Adjusted HRs for common cancers associated with heavy episodic drinking, by total weekly amount in male current regular drinkers 26

Figure S11. Adjusted HRs for common cancers associated with duration of regular drinking, in male current regular drinkers 27

Figure S12. Adjusted HRs per 280 g/week higher usual alcohol intake for common cancers, by flushing status in male current regular drinkers 28

Table S12. Adjusted HRs per 280 g/week higher usual alcohol intake for incident cancers, in male current regular drinkers – in sequentially adjusted models 29

Figure S13. Associations of alcohol consumption with IARC alcohol-related cancers, other cancers, and total cancer, in all men 30

Figure S14. Associations of alcohol consumption with common cancers in all men 31

Figure S15. Associations of alcohol consumption with other site-specific cancers, in all men 32

Figure S16. Associations of alcohol consumption with other less common site-specific cancers, in all men 33

Table S13. Adjusted HRs for incident cancers associated with alcohol drinking status, after various exclusions of participants in men 34

Table S14. Adjusted HRs for incident cancers associated with level of alcohol consumption, after various exclusions of participants in male current regular drinkers 35

Table S15. Population attributable fraction of ever-regular drinking on incidence of common cancers 36

Table S16. Adjusted HRs for cancer mortality associated with alcohol drinking status, in men 37

Table S17. Adjusted HRs for cancer mortality associated with level of alcohol consumption, in male current regular drinkers 38

Table S18. Reproducibility of self-reported alcohol consumption data in the CKB 39

Table S19. Prevalence of alcohol consumption at baseline (2004-2008), first (2008) and second resurveys (2013-2014), by sex 40

Table S20. Patterns of alcohol consumption at baseline (2004-2008), first (2008) and second resurveys (2013-2014), among current regular drinkers, by sex 41

Table S21. Associations of blood pressure and gamma-glutamyl transferase with alcohol consumption among men at baseline 42

References 43

Members of the China Kadoorie Biobank collaborative group

International Steering Committee: Junshi Chen, Zhengming Chen (PI), Robert Clarke, Rory Collins, Yu Guo, Liming Li (PI), Jun Lv, Richard Peto, Robin Walters.

International Co-ordinating Centre, Oxford: Daniel Avery, Ruth Boxall, Derrick Bennett, Yumei Chang, Yiping Chen, Zhengming Chen, Robert Clarke, Huaidong Du, Simon Gilbert, Alex Hacker, Parisa Hariri, Michael Holmes, Andri Iona, Christiana Kartsonaki, Rene Kerosi, Garry Lancaster, Kuang Lin, John McDonnell, Iona Millwood, Qunhua Nie, Richard Peto, Paul Ryder, Sam Sansome, Dan Schmidt, Rajani Sohoni, Becky Stevens, Iain Turnbull, Robin Walters, Lin Wang, Neil Wright, Ling Yang, Xiaoming Yang.

National Co-ordinating Centre, Beijing: Zheng Bian, Ge Chen, Yu Guo, Xiao Han, Can Hou, Chao Liu, Pei Pei, Shuzhen Qu, Yunlong Tan, Canqing Yu.

10 Regional Co-ordinating Centres: Qingdao Qingdao CDC: Zengchang Pang, Ruqin Gao, Shanpeng Li, Shaojie Wang, Yongmei Liu, Ranran Du, Yajing Zang, Liang Cheng, Xiaocao Tian, Hua Zhang, Yaoming Zhai, Feng Ning, Xiaohui Sun, Feifei Li. Licang CDC: Silu Lv, Junzheng Wang, Wei Hou. **Harbin** Heilongjiang Provincial CDC: Mingyuan Zeng, Ge Jiang, Xue Zhou. Nangang CDC: Liqiu Yang, Hui He, Bo Yu, Yanjie Li, Qinai Xu, Quan Kang, Ziyan Guo. **Haikou** Hainan Provincial CDC: Dan Wang, Ximin Hu, Jinyan Chen, Yan Fu, Zhenwang Fu, Xiaohuan Wang. Meilan CDC: Min Weng, Zhendong Guo, Shukuan Wu, Yilei Li, Huimei Li, Zhifang Fu. **Suzhou** Jiangsu Provincial CDC: Ming Wu, Yonglin Zhou, Jinyi Zhou, Ran Tao, Jie Yang, Jian Su. Suzhou CDC: Fang Liu, Jun Zhang, Yihe Hu, Yan Lu, Liangcai Ma, Aiyu Tang, Shuo Zhang, Jianrong Jin, Jingchao Liu. **Liuzhou** Guangxi Provincial CDC: Zhenzhu Tang, Naying Chen, Ying Huang. Liuzhou CDC: Mingqiang Li, Jinhuai Meng, Rong Pan, Qilian Jiang, Jian Lan, Yun Liu, Liuping Wei, Liyuan Zhou, Ningyu Chen, Ping Wang, Fanwen Meng, Yulu Qin, Sisi Wang. **Sichuan** Sichuan Provincial CDC: Xianping Wu, Ningmei Zhang, Xiaofang Chen, Weiwei Zhou. Pengzhou CDC: Guojin Luo, Jianguo Li, Xiaofang Chen, Xunfu Zhong, Jiaqiu Liu, Qiang Sun. **Gansu** Gansu Provincial CDC: Pengfei Ge, Xiaolan Ren, Caixia Dong. Maiji CDC: Hui Zhang, Enke Mao, Xiaoping Wang, Tao Wang, Xi Zhang. **Henan** Henan Provincial CDC: Ding Zhang, Gang Zhou, Shixian Feng, Liang Chang, Lei Fan. Huixian CDC: Yulian Gao, Tianyou He, Huarong Sun, Pan He, Chen Hu, Xukui Zhang, Pan He, Huifang Wu. **Zhejiang** Zhejiang Provincial CDC: Min Yu, Ruying Hu, Hao Wang. Tongxiang CDC: Yijian Qian, Chunmei Wang, Kaixu Xie, Lingli Chen, Yidan Zhang, Dongxia Pan, Qijun Gu. **Hunan** Hunan Provincial CDC: Yuelong Huang, Biyun Chen, Li Yin, Huilin Liu, Zhongxi Fu, Qiaohua Xu. Liuyang CDC: Xin Xu, Hao Zhang, Huajun Long, Xianzhi Li, Libo Zhang, Zhe Qiu.

Supplementary Methods

Participant exclusion

The analyses excluded participants with a self-reported history of cancer and, where relevant, chronic diseases which are risk factors of specific cancers[1, 2] to avoid reverse causality and potential bias by changes in alcohol drinking behaviours related to prior diseases. Participants with a self-reported history of cancer at baseline (n=2578) were excluded from all analyses in the present study. Participants with a self-reported history of liver cirrhosis or chronic hepatitis (n=6139) were further excluded from analyses of liver cancer, while those with a self-reported history of bronchitis or emphysema (n=13,231), tuberculosis (n=7019), or chronic obstructive pulmonary disease (n=22,945 including self-reported and screen-detected cases from spirometry) were excluded from analyses of lung cancer.

Assessment of smoking

Detailed information related to smoking including frequency, amount, type of tobacco smoked, the age at which participants started smoking regularly and stopped smoking, and the main reason for smoking cessation were collected by baseline questionnaire. Based on a combination of these information, for the present study, participants were categorised into: "never-regular smokers" (including never smokers [did not smoke at baseline and had smoked <100 cigarettes in lifetime] and occasional smokers [did not smoke at baseline but had smoked occasionally in the past or had smoked at least 100 cigarettes in lifetime, or only smoked occasionally at baseline but had never smoked on most days or daily in lifetime]); and "ever-regular smokers" (including ex-regular and current regular smokers; i.e. ever smoked regularly [on most days or daily]). Exhaled carbon monoxide (COex) was measured to help validate exposure to smoking (CareFusion MicroCOmeter).[3] Previous reports in CKB showed a much higher mean COex level in current regular smokers (which further increased with intensity and duration of smoking) than in participants of other smoking categories (i.e., never smokers, ex-regular smokers, and occasional smokers; among whom the COex level was similar),[3] and clear elevated tobacco-attributed mortality and lung cancer risk among ex-smokers and regular smokers (which further increased with intensity and duration of smoking) compared with non-smokers,[4, 5] suggesting good validity of self-reported smoking data in CKB.

Statistical models

Among current regular drinkers, males were grouped into four consumption categories (<140, 140-279, 280-419, 420+ g/week), and females into three categories (<70, 70-139, 140+ g/week). Cox regression models were fitted to estimate the association of alcohol drinking status (reference group: abstainers) with incident cancers in all men and women separately, and alcohol consumption level (in categories [reference group: <140 g/week for men; <70 g/week for women] and as a continuous variable [per 280 g/week increment]) to incident cancers in male current regular drinkers and female current regular drinkers respectively. Cox regression models were stratified by age-at-risk (five-year groups) and study area (ten areas), and were adjusted for education (no formal school, primary school, middle or high school, technical school/college or above), household income (<10 000, 10 000-19 999, 20 000-34 999, 35 000+ yuan/year), smoking (five groups in men: never, occasional, ever regular <15, ever regular 15-24, ever regular 25+ cigarettes equivalent/day; four groups in women: never, occasional, ex-regular, current), physical activity (<17.8, 17.8-28.7, 28.8+ metabolic equivalent of task hours [MET-h] per day), fruit intake (daily vs. less than daily), BMI (<22, 22-24.9, 25-26.9, 27+ kg/m^2), and family history of cancer (yes/no). These covariates were selected based on their relationships with cancer and their correlations with alcohol drinking behaviours reported in existing literature and in the CKB.[6-9] Further adjustment for total weekly consumption as a continuous variable was conducted in the analyses of drinking patterns (daily drinking, HED, mealtime habit, spirit drinking, duration of regular drinking), and baseline age for analyses of duration of regular drinking. For daily drinking and HED, the risk estimates were further examined across subgroups by total weekly intake (<280 vs. ≥280 g/week). For analyses involving comparisons of just two groups (i.e. an exposure category with the reference group), conventional 95% CIs

were reported. For analyses involving more than two categories of exposure, floating SEs were used to estimate group-specific 95% CIs of the log HRs of all categories including the reference group, enabling comparison between any two categories rather than just pairwise comparisons with the reference group.[10] Departure from linearity was assessed using restricted cubic splines with five knots at the 5th, 27.5th, 50th, 72.5th and 95th percentiles of the total distribution of alcohol intake. Likelihood ratio tests, which compared the model with cubic spline terms including the linear term versus the model with only the linear term, indicated evidence of non-linear associations for oesophageal cancer ($p_{non-linearity}<0.0001$), brain cancer ($p_{non-linearity}=0.027$), IARC alcohol-related cancers ($p_{non-linearity}=0.003$), other cancers of known sites ($p_{non-linearity}=0.002$), and total cancers ($p_{non-linearity}=0.006$), with no strong evidence for other site-specific cancers ($p_{non-linearity}=0.11$-0.93).

Adjustment for regression dilution bias

A gradual regression to the mean over time was observed for alcohol consumption in the subset of participants involved in all three CKB surveys (**Table S3**). Within-person variation of self-reported alcohol intake was addressed using the regression dilution approach,[11] whereby the usual alcohol intake in each baseline consumption category was taken to be the average intake of the two resurveys in 2008 and 2013-2014, assuming that occasional drinkers consumed 5 g/week. The HRs of baseline consumption categories were plotted against their corresponding mean usual alcohol intake. The regression dilution ratio (RDR) was calculated using the assumption-free, non-parametric McMahon-Peto method,[12] taken as the ratio of the range (i.e. difference in the mean alcohol intake of the top [i.e. 420+ g/week for men; 140+ g/week for women] vs. bottom [i.e. <140 g/week for men; <70 g/week for women] baseline-defined groups) of the usual alcohol intake to the range of baseline alcohol intake. The RDRs calculated using the McMahon-Peto method were 0.54 for men and 0.56 for women, broadly similar to the estimates obtained from other methods, e.g. self-correlation[11] and the Rosner's regression method.[13] Log HR estimates and corresponding standard errors for baseline alcohol intake, modelled as a continuous variable, were then divided by the RDR calculated from the McMahon-Peto method to obtain estimated HRs per 280 g/week higher usual alcohol intake among current regular drinkers, assuming a linear association. The HR per 100 g/week is approximately the cube root of the HR per 280 g/week (as log HR per 100 g/week is [100/280] times log HR per 280 g/week).

Table S1. Definitions of main alcohol drinking patterns

Parameters	Description
**Drinking status**	
Abstainers	Past 12 months: Never drank alcohol.<br>In the past: Had not drunk weekly or more frequently.
Ex-regular drinkers	Past 12 months: Never drank alcohol; or had drunk alcohol occasionally, at certain seasons, or monthly but less than weekly.<br>In the past: Had drunk at least weekly.
Occasional drinkers	Past 12 months: Had drunk alcohol occasionally, at certain seasons, or monthly but less than weekly.<br>In the past: Had not drunk weekly or more frequently.
Current regular drinkers	Past 12 months: At least weekly (i.e. drank alcohol in most weeks).<br>In the past: N/A.
**Drinking patterns**	
Drinking frequency	Daily: 6-7 days/week (i.e. daily or almost every day).<br>Non-daily: 1-2 days/week or 3-5 days/week.
Mean consumption[a]	Calculated based on the beverage type, amount drunk (g of pure alcohol/session), and drinking frequency.<br>Alcohol content by volume (v/v) of each beverage type are assumed as the following:[14] beer 4%, grape wine 12%, rice wine 15%, weak spirits 38%, and strong spirits 53%.
Types of alcohol[a]	Spirits: Strong spirit (≥40% alcohol) or weak spirit (<40% alcohol).<br>Non-spirits: Rice wine, grape wine or beer.
Heavy episodic drinking[a]	Men: Consumption of >60 g (i.e. 7.5 UK units or 4.3 US standard drinks) of alcohol per session.<br>Women: Consumption of >40 g (i.e. 5 UK units or 2.9 US standard drinks) of alcohol per session.[15]
Flushing response	Experiencing hot flushes or dizziness soon after first mouthful or after drinking small amount of alcohol.
Drinking with/outside of meals	With meals: Usually drank with meals.<br>Outside of meals: Usually drank between or after meals or no regular patterns.
Duration of regular drinking	Number of years of regular drinking calculated by the difference between baseline age (years) and age started regular drinking (years).

[a] Data was available on a typical drinking day, on special occasions, and the last time the participants drank. Alcohol data reported on a typical drinking day was used in the analyses to reflect the usual drinking habits of the participants.

Table S2. Number of incident cancer cases reported in men and women without prior cancer in the CKB

Cancer site (ICD-10 code)	Number of cases	
	Men	Women
Mouth and throat (C00-C14, C32)	541	312
Oesophagus (C15)	1608	740
Colon and rectum (C18-C20)	1527	1529
Colon (C18)	856	901
Rectum (C19-C20)	946	883
Liver (C22)[a]	1651	975
Female breast (C50)	--	2289
Stomach (C16)	2221	1123
Pancreas (C25)	405	399
Lung (including trachea) (C33-C34)[b]	2741	2020
Gallbladder, other and unspecified parts of biliary tract (C23-C24)	279	356
Skin cancer (C43-C44)	137	130
Cervix uteri (C53)	--	1054
Corpus uteri (C54)	--	492
Endometrium (C54.1)	--	410
Uterus (C55)	--	333
Ovary (C56)	--	433
Prostate (C61)	402	--
Kidney (C64)	219	204
Bladder (C67)	356	164
Brain (C71)	227	270
Thyroid (C73)	84	461
Lymphoma (C81-C85)	402	421
Multiple myeloma and malignant plasma cell neoplasms (C90)	137	114
Leukaemia (C91-C95)	287	321
Other less common cancers of known sites	935	925
IARC alcohol-related cancers (C00-C15, C32, C18-C20, C22, C50)	5403	5754
Other cancers of known sites (Non-IARC alcohol-related)	8730	8318
All cancers[c]	13342	13619

ICD-10, International Classification of Diseases, 10th revision; IARC, International Agency for Research on Cancer.
Participants with prior cancer were excluded.
[a] Further excluded prior liver cirrhosis or chronic hepatitis.
[b] Further excluded prior tuberculosis, emphysema or bronchitis, and chronic obstructive pulmonary disease (both self-reported and screen-detected).
[c] All cancers included ill-defined neoplasm and are patient-based.

Table S3. Estimated regression dilution ratios of alcohol intake in men and women

		Mean consumption, g/week				Regression dilution ratio[a]			
Baseline-defined groups	N	Baseline	1st resurvey	2nd resurvey	Usual	MacMahon-Peto method	Pearson's correlation	Spearman's correlation	Rosner's regression
**Men**									
Abstainers & ex-regular drinkers	1255	0.0	8.3	13.1	10.7				
Occasional drinkers	2464	5.0	22.9	39.4	31.2				
Current regular drinkers									
<140 g/week	696	77.8	96.1	118.5	107.3				
140-279 g/week	487	223.9	187.7	240.7	214.2				
280-419 g/week	336	367.6	294.8	305.3	300.1				
420+ g/week	380	686.1	440.9	435.5	438.2				
Regression dilution ratio[a]						0.54	0.52	0.55	0.55
**Women**									
Abstainers & ex-regular drinkers	5897	0.0	0.9	0.8	0.9				
Occasional drinkers	3098	5.0	4.5	3.8	4.2				
Current regular drinkers									
<70 g/week	88	33.4	28.7	19.9	24.3				
70-139 g/week	58	115.0	93.6	54.7	74.1				
140+ g/week	59	318.1	201.9	167.6	184.7				
Regression dilution ratio[a]						0.56	0.59	0.49	0.69

Participants with prior cancer were excluded.

Usual consumption is the average alcohol intake of the two resurveys.

[a] Regression dilution ratio is estimated among baseline current regular drinkers using baseline and usual alcohol intakes. The regression dilution ratios calculated using the MacMahon-Peto method based on baseline survey and first resurvey are 0.57 for men and 0.61 for women, and that based on baseline and second resurvey is 0.52 for both men and women.

Table S4. Baseline characteristics of participants by alcohol drinking categories, in women

	Overall		Abstainers		Ex-regular drinkers		Occasional drinkers		Current regular drinkers							
									All current regular		<70 g/week		70-139 g/week		140+ g/week	
Number of participants	300900		191272		2630		100779		6219		3203		1585		1431	
**Socio-demographic characteristics**																
Mean age, years (SD)	51.4	(10.5)	52.6	(10.7)	56.6	(9.4)	49.1	(9.9)	52.5	(10.3)	53.2	(10.7)	53.2	(10.1)	52.8	(9.5)
Urban area, %	44.5		42.6		30.0		48.0		46.4		61.4		35.5		26.0	
Educational attainment >6 years, %	43.3		41.2		46.9		49.0		48.2		50.0		44.6		47.7	
Income >20000 yuan/year, %	40.7		38.0		45.7		44.2		46.9		49.5		44.2		46.3	
Married, %	89.0		88.8		84.3		89.3		87.8		88.2		87.8		85.9	
**Lifestyle factors**																
Regular smoking, %	2.4		1.9		5.4		2.8		7.9		5.5		8.2		16.0	
Daily fresh fruit consumption, %	68.3		70.1		57.0		63.1		60.9		57.5		65.7		67.2	
Physical activity, mean MET-h/d (SD)	20.5	(12.8)	20.2	(13.3)	20.3	(11.1)	20.8	(11.7)	21.2	(11.6)	20.2	(11.5)	19.7	(11.8)	19.9	(11.5)
Daily tea drinking, %	15.9		14.8		25.4		18.9		30.8		29.1		34.3		32.7	
**Physical measurements, mean (SD)**																
Body mass index, kg/m^2	23.8	(3.5)	23.9	(3.5)	24.0	(3.5)	23.7	(3.4)	23.6	(3.4)	23.7	(3.4)	23.6	(3.3)	23.7	(3.4)
Systolic blood pressure, mmHg	129.9	(22.0)	130.9	(22.5)	130.4	(23.2)	128.0	(20.5)	128.6	(21.6)	127.4	(20.8)	128.4	(21.8)	129.9	(22.5)
Diastolic blood pressure, mmHg	76.8	(10.9)	77.1	(11.0)	77.5	(11.2)	76.0	(10.6)	77.0	(10.9)	76.3	(10.7)	77.2	(10.9)	77.9	(11.3)
**Health and medical history, %[a]**																
Poor health	11.3		12.5		21.5		9.5		7.9		7.7		7.8		8.7	
Any chronic disease[b]	21.7		22.7		31.8		20.5		19.3		20.6		18.4		18.0	
Coronary heart disease	3.2		3.5		5.4		2.7		2.6		2.6		2.6		1.8	
Stroke/transient ischaemic attack	1.3		1.6		2.6		1.0		0.7		0.7		0.6		1.0	
Liver cirrhosis/chronic hepatitis	0.8		0.9		1.8		0.8		0.8		0.8		0.4		1.4	
Emphysema/bronchitis	2.2		2.3		2.4		2.2		1.8		1.7		1.0		3.2	
Chronic obstructive pulmonary disease	6.2		6.4		9.6		5.9		5.7		5.3		5.2		7.0	
Diabetes	6.1		7.0		8.0		4.9		3.5		3.8		2.8		4.5	
Family history of cancer	16.3		15.6		20.8		17.5		18.7		18.9		16.6		18.8	

SD, standard deviation; MET-h/d, metabolic equivalents of task per hours per day.
Participants were prior cancer were excluded.
Prevalences and means are adjusted for age and study areas as appropriate.
[a] All self-reported except for chronic obstructive pulmonary disease and diabetes which included both self-reported and screen-detected events.
[b] Chronic diseases included self-reported coronary heart disease, stroke, transient ischaemic attack, diabetes, tuberculosis, chronic hepatitis/liver cirrhosis, rheumatoid arthritis, peptic ulcer, emphysema/bronchitis, gallstone/gallbladder disease, and kidney disease.

Table S5. Baseline drinking characteristics of participants by level of alcohol consumption, in male current regular drinkers

	All current regular		Weekly consumption							
			<140 g/week		140-279 g/week		280-419 g/week		420+ g/week	
Number of participants	69734		24999		18874		12811		13050	
**Alcohol drinking characteristics**										
Mean alcohol consumption, g/week (SD)	285.9	(245.3)	80.8	(38.9)	221.8	(44.9)	371.5	(47.1)	687.3	(237.1)
Daily drinking, %	62.1		31.4		65.2		82.1		94.6	
Heavy episodic drinking on typical day, %	37.2		9.9		14.4		63.3		100.0	
Drinking spirits on typical day, %	69.6		53.6		71.0		82.4		87.4	
Drinking with meals, %	85.9		86.3		86.5		86.0		83.3	
Flushing response, %	17.9		26.7		17.5		14.5		9.2	
Mean years of regular drinking, year (SD)	22.9	(12.4)	20.9	(12.7)	23.0	(12.3)	24.2	(11.7)	25.6	(11.4)
Mean age started regular drinking, year (SD)	28.6	(11.0)	30.7	(12.2)	28.7	(10.6)	27.3	(9.7)	26.0	(9.0)

SD, standard deviation.
Participants with prior cancer were excluded.
Prevalences and means are adjusted for age and study areas as appropriate.

Table S6. Baseline drinking characteristics of participants by level of alcohol drinking, in female current regular drinkers

	All current regular		Weekly consumption					
			<70 g/week		70-139 g/week		140+ g/week	
Number of participants	6219		3203		1585		1431	
**Alcohol drinking characteristics**								
Mean alcohol consumption, g/week (SD)	115.8	(127.4)	39.0	(19.1)	109.3	(23.8)	294.8	(139.0)
Daily drinking, %	45.2		22.2		59.9		70.3	
Heavy episodic drinking on typical day, %	26.5		9.7		9.8		79.8	
Drinking spirits on typical day, %	61.9		56.6		66.4		81.9	
Drinking with meals, %	86.3		86.6		85.4		88.1	
Flushing response, %	23.6		26.7		22.9		19.8	
Mean years of regular drinking, years (SD)	15.4	(12.9)	13.6	(11.7)	16.1	(12.7)	19.0	(13.0)
Mean age started regular drinking, years (SD)	37.7	(13.5)	39.5	(13.6)	37.1	(13.1)	34.1	(12.0)

SD, standard deviation.
Participants with prior cancer were excluded.
Prevalences and means are adjusted for age and study areas as appropriate.

Table S7. Adjusted HRs for incident cancers associated with drinking status, in women

Cancer site	All women N	Abstainers N	Abstainers HR (95% CI)	Ex-regular N	Ex-regular HR (95% CI)	Occasional N	Occasional HR (95% CI)	Current regular N	Current regular HR (95% CI)	*P*[a]
Mouth and throat	312	214	1.00 (0.83-1.20)	4	1.23 (0.45-3.33)	89	1.11 (0.89-1.38)	5	0.73 (0.30-1.78)	0.52
Oesophagus	740	340	1.00 (0.89-1.13)	8	1.17 (0.57-2.41)	377	0.99 (0.88-1.12)	15	1.23 (0.73-2.06)	0.48
Colon and rectum	1529	1018	1.00 (0.92-1.08)	17	0.92 (0.57-1.50)	453	0.96 (0.87-1.06)	41	1.10 (0.81-1.51)	0.58
Colon	901	600	1.00 (0.90-1.11)	8	0.70 (0.35-1.42)	265	0.93 (0.82-1.05)	28	1.21 (0.83-1.76)	0.36
Rectum	883	587	1.00 (0.90-1.11)	11	0.96 (0.53-1.76)	265	1.02 (0.90-1.16)	20	0.95 (0.61-1.48)	0.80
Liver	975	702	1.00 (0.91-1.10)	16	1.17 (0.71-1.94)	236	0.78 (0.68-0.89)	21	0.81 (0.52-1.25)	0.35
Breast	2289	1280	1.00 (0.93-1.08)	19	1.24 (0.79-1.95)	934	1.12 (1.05-1.20)	56	1.16 (0.89-1.52)	0.28
Stomach	1123	788	1.00 (0.91-1.10)	7	0.61 (0.29-1.30)	304	0.96 (0.85-1.08)	24	0.99 (0.66-1.49)	0.96
Pancreas	399	274	1.00 (0.86-1.17)	5	0.94 (0.38-2.29)	108	0.96 (0.79-1.17)	12	1.10 (0.62-1.97)	0.75
Lung	2020	1344	1.00 (0.93-1.07)	26	1.05 (0.71-1.55)	610	0.96 (0.89-1.05)	40	0.67 (0.49-0.92)	0.02
Gallbladder	356	261	1.00 (0.85-1.18)	2	0.41 (0.10-1.64)	82	0.98 (0.78-1.24)	11	1.30 (0.71-2.38)	0.43
Skin	130	87	1.00 (0.75-1.33)	1	0.63 (0.09-4.61)	40	1.58 (1.14-2.21)	2	0.71 (0.17-2.88)	0.65
Cervix uteri	1054	719	1.00 (0.91-1.10)	14	1.61 (0.94-2.74)	303	0.99 (0.87-1.11)	18	0.98 (0.62-1.57)	0.91
Corpus uteri	492	324	1.00 (0.87-1.16)	3	0.67 (0.21-2.09)	158	0.86 (0.73-1.01)	7	0.63 (0.30-1.33)	0.24
Endometrium	410	258	1.00 (0.85-1.17)	3	0.85 (0.27-2.66)	143	0.89 (0.75-1.06)	6	0.62 (0.28-1.40)	0.28
Uterus	333	220	1.00 (0.84-1.19)	4	1.22 (0.45-3.29)	102	0.93 (0.75-1.15)	7	0.99 (0.47-2.10)	0.997
Ovary	433	282	1.00 (0.86-1.17)	4	1.03 (0.38-2.79)	139	0.86 (0.72-1.03)	8	0.82 (0.41-1.65)	0.58
Kidney	204	125	1.00 (0.79-1.26)	3	1.80 (0.56-5.72)	72	1.10 (0.86-1.40)	4	0.83 (0.31-2.23)	0.71
Bladder	164	106	1.00 (0.79-1.27)	4	2.31 (0.84-6.36)	52	1.28 (0.96-1.72)	2	0.55 (0.14-2.22)	0.39
Brain	270	194	1.00 (0.82-1.21)	2	0.63 (0.16-2.56)	66	0.73 (0.56-0.94)	8	1.08 (0.53-2.20)	0.79
Thyroid	461	271	1.00 (0.85-1.18)	6	1.82 (0.81-4.11)	172	0.93 (0.79-1.09)	12	1.01 (0.57-1.79)	0.996
Lymphoma	421	297	1.00 (0.86-1.17)	6	1.22 (0.54-2.77)	112	1.01 (0.83-1.24)	6	0.68 (0.30-1.52)	0.35
Multiple myeloma and malignant plasma cell neoplasms	114	76	1.00 (0.76-1.32)	2	1.32 (0.32-5.47)	35	1.11 (0.78-1.57)	1	0.41 (0.06-2.94)	0.35
Leukaemia	321	226	1.00 (0.84-1.19)	2	0.58 (0.14-2.33)	84	0.83 (0.66-1.04)	9	1.22 (0.63-2.38)	0.56
IARC alcohol-related cancers	5754	3502	1.00 (0.96-1.04)	63	1.14 (0.88-1.46)	2054	1.01 (0.96-1.06)	135	1.05 (0.89-1.25)	0.59
Other cancers of known sites (Non-IARC alcohol-related)	8318	5625	1.00 (0.97-1.04)	96	0.98 (0.80-1.20)	2417	0.94 (0.90-0.98)	180	0.85 (0.74-0.99)	0.04
All cancers[b]	13619	8829	1.00 (0.97-1.03)	155	1.05 (0.90-1.24)	4334	0.97 (0.94-1.00)	301	0.92 (0.82-1.03)	0.14

HR, hazard ratio; CI, confidence interval; IARC, International Agency for Research on Cancer.

Cox models are stratified by age-at-risk and study area, and adjusted for education, income, smoking, physical activity, fruit intake, body mass index, and family history of cancer.

Participants with self-reported prior cancer were excluded from all analyses. Participants with self-reported prior chronic hepatitis/liver cirrhosis were further excluded from analysis of liver cancer, and participants with self-reported prior tuberculosis, emphysema/bronchitis, or chronic obstructive pulmonary disease were further excluded from analysis of lung cancer.

[a] Two-sided *P* values for association comparing current regular drinkers vs. abstainers.

[b] All cancers included ill-defined neoplasm and are patient-based.

Figure S1. Associations of alcohol consumption with incident cancers, in female current regular drinkers

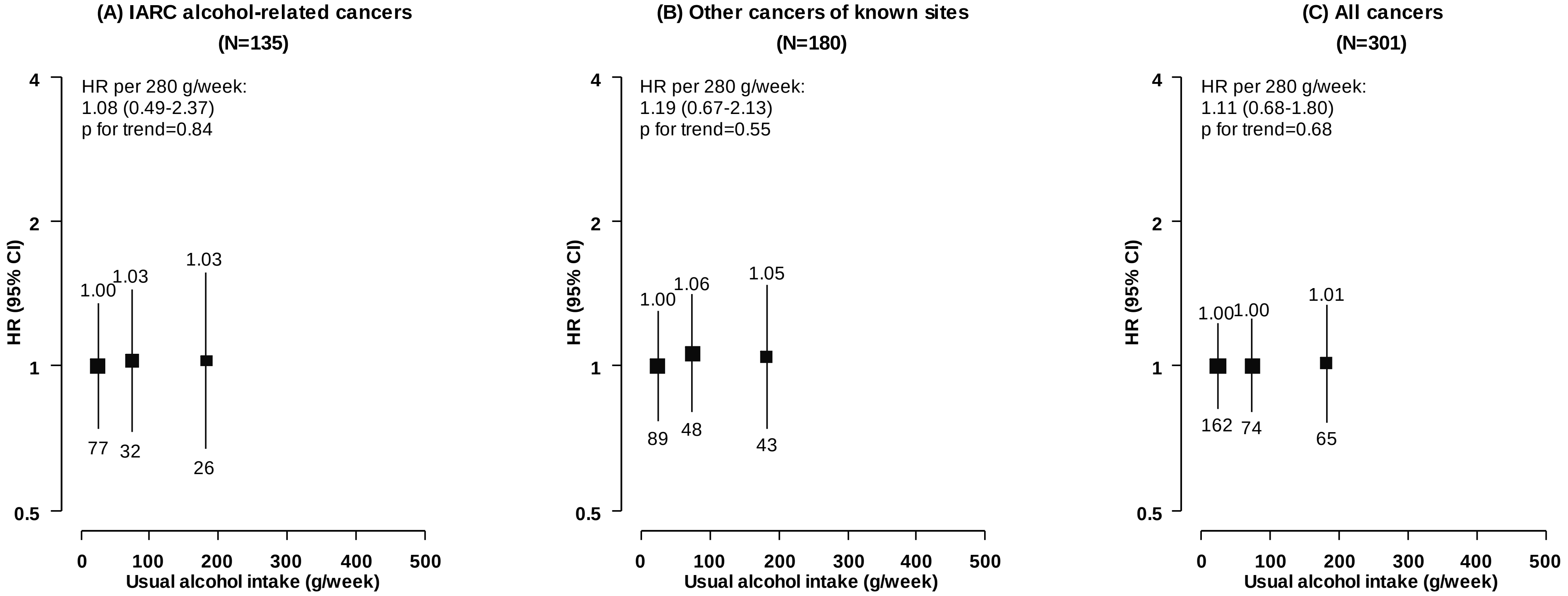

Cox models are stratified by age-at-risk and study area, and adjusted for education, income, smoking status, physical activity, fresh fruit intake, body mass index, and family history of cancer. Each solid square represents an HR. The size of each box is inversely proportional to the "floated" variance of the log HR in each group and the error bars indicate the group-specific 95% CI. The numbers above the error bars are point estimates for HRs, and the numbers below are number of events. Usual alcohol intake is calculated by the average of the self-reported alcohol intakes at two resurveys in each group. *P* for trend is estimated by modelling alcohol consumption (g/week) as a continuous variable among current regular drinkers. HR, hazard ratio; CI, confidence interval; IARC, International Agency for Research on Cancer.

Table S8. Adjusted HRs for incident cancers associated with level of alcohol consumption, in female current regular drinkers

Cancer site	All current regular N	<70 g/week		70+ g/week		
		N	HR (95% CI)	N	HR (95% CI)	*P*
Oesophagus	15	6	1.00 (Reference)	9	1.65 (0.48-5.71)	0.43
Colon and rectum	41	22	1.00 (Reference)	19	1.25 (0.60-2.63)	0.55
Colon	28	15	1.00 (Reference)	13	1.04 (0.42-2.59)	0.94
Rectum	20	10	1.00 (Reference)	10	1.86 (0.61-5.66)	0.27
Liver	21	8	1.00 (Reference)	13	1.39 (0.48-3.97)	0.54
Breast	56	40	1.00 (Reference)	16	0.68 (0.36-1.30)	0.24
Stomach	24	7	1.00 (Reference)	17	2.67 (0.96-7.46)	0.06
Pancreas	12	6	1.00 (Reference)	6	0.78 (0.22-2.80)	0.70
Lung	40	20	1.00 (Reference)	20	0.91 (0.44-1.87)	0.80
Gallbladder	11	5	1.00 (Reference)	6	0.73 (0.19-2.72)	0.64
Cervix uteri	18	11	1.00 (Reference)	7	0.65 (0.22-1.94)	0.44
Thyroid	12	8	1.00 (Reference)	4	1.27 (0.35-4.56)	0.71
IARC alcohol-related cancers	135	77	1.00 (Reference)	58	1.00 (0.67-1.49)	0.99
Other cancers of known sites (Non-IARC alcohol-related)	180	89	1.00 (Reference)	91	1.05 (0.75-1.46)	0.79
All cancers[a]	301	162	1.00 (Reference)	139	0.99 (0.76-1.29)	0.94

HR, hazard ratio; CI, confidence interval; IARC, International Agency for Research on Cancer.

Cox models are stratified by age-at-risk and study area, and adjusted for education, income, smoking, physical activity, fruit intake, body mass index, and family history of cancer.

Participants with self-reported prior cancer were excluded from all analyses. Participants with self-reported prior chronic hepatitis/liver cirrhosis were further excluded from analysis of liver cancer, and participants with self-reported prior tuberculosis, emphysema/bronchitis, or chronic obstructive pulmonary disease were further excluded from analysis of lung cancer.

[a] All cancers included ill-defined neoplasm and are patient-based.

Figure S2. Associations of alcohol consumption with colon cancer and rectal cancer, in male current regular drinkers

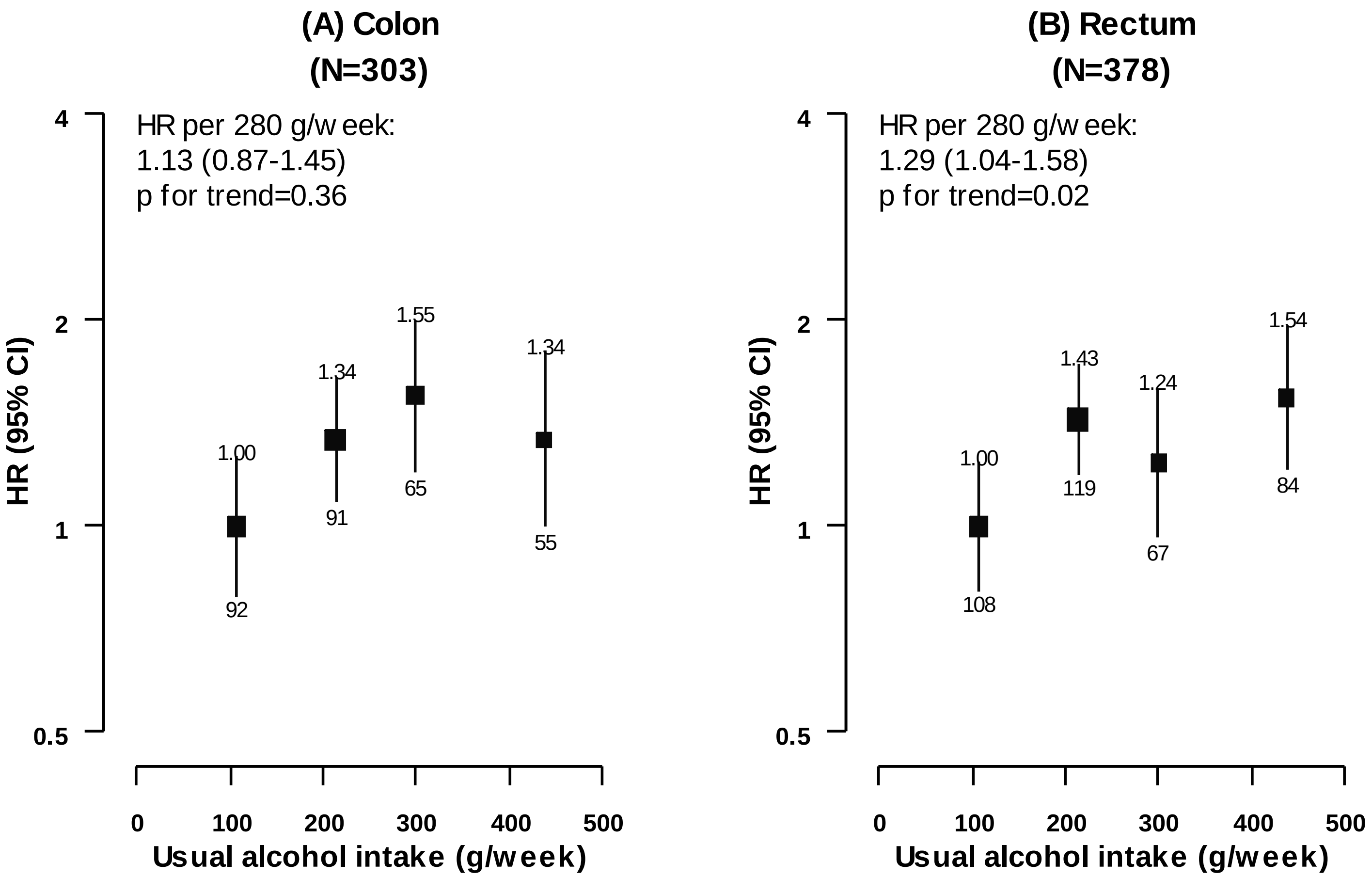

Conventions are as in Figure S1.

Table S9. Adjusted HRs for lip and oral cavity cancer, pharyngeal cancer, and laryngeal cancer associated with alcohol drinking, in men

		Abstainers		Ex-regular drinkers		Occasional drinkers		Current regular drinkers				
Cancer site	All men N	N	HR (95% CI)	N	HR (95% CI)	N	HR (95% CI)	N	HR (95% CI)	P^{a}	HR (95% CI) per 280 g/week	P_{trend}^{b}
Lip and oral cavity	140	23	1.00 (0.65-1.53)	12	1.06 (0.60-1.87)	39	1.33 (0.96-1.86)	66	1.89 (1.46-2.45)	0.01	2.04 (1.45-2.87)	<0.001
Pharynx (excluding nasopharynx)	67	10	1.00 (0.53-1.89)	9	1.81 (0.93-3.50)	15	1.18 (0.69-2.00)	33	2.05 (1.42-2.96)	0.06	1.71 (1.11-2.66)	0.02
Larynx	164	18	1.00 (0.62-1.61)	19	2.05 (1.30-3.23)	36	1.50 (1.07-2.11)	91	3.30 (2.64-4.13)	<0.001	2.00 (1.56-2.56)	<0.001

HR, hazard ratio; CI, confidence interval; IARC, International Agency for Research on Cancer.
Cox models are stratified by age-at-risk and study area, and adjusted for education, income, smoking, physical activity, fruit intake, body mass index, and family history of cancer.
Participants with self-reported prior cancer were excluded from all analyses.
[a] *P* value for association comparing current regular drinkers vs. abstainers.
[b] *P* value for alcohol consumption (g/week) modelled as a continuous variable among current regular drinkers.

Figure S3. Associations of alcohol consumption with lip and oral cavity cancer, pharyngeal cancer, and laryngeal cancer, in male current regular drinkers

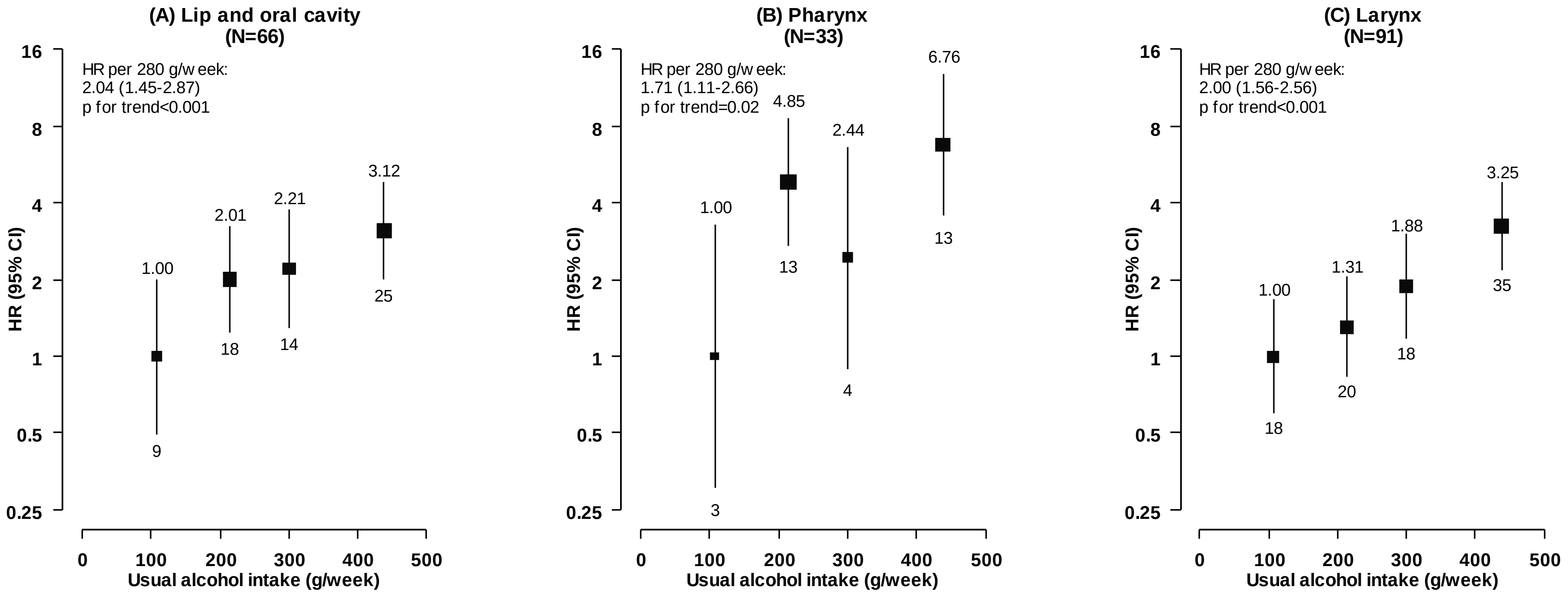

Conventions are as in Figure S1.

Figure S4. Associations of alcohol consumption with lung cancer, by smoking status in male current regular drinkers

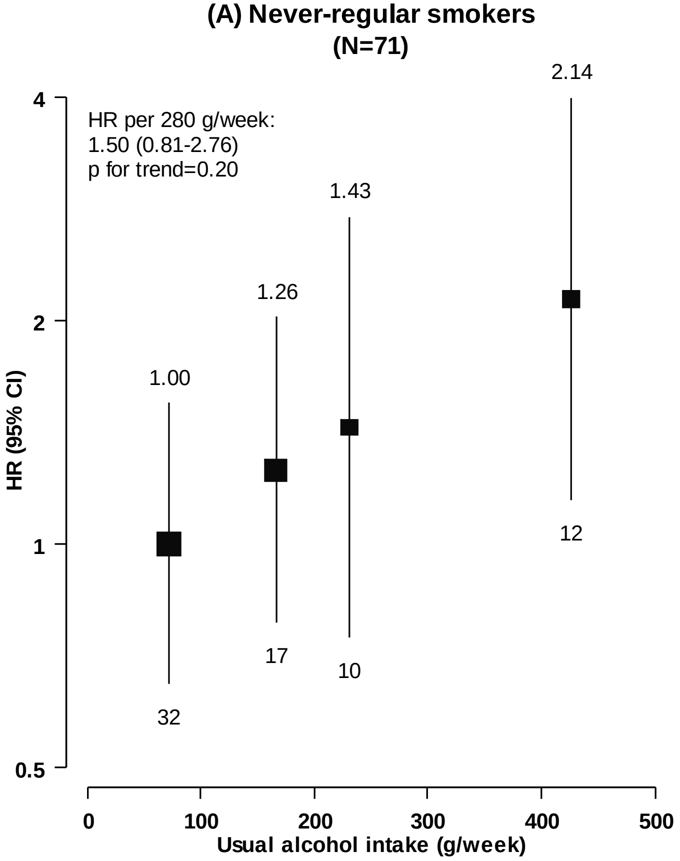

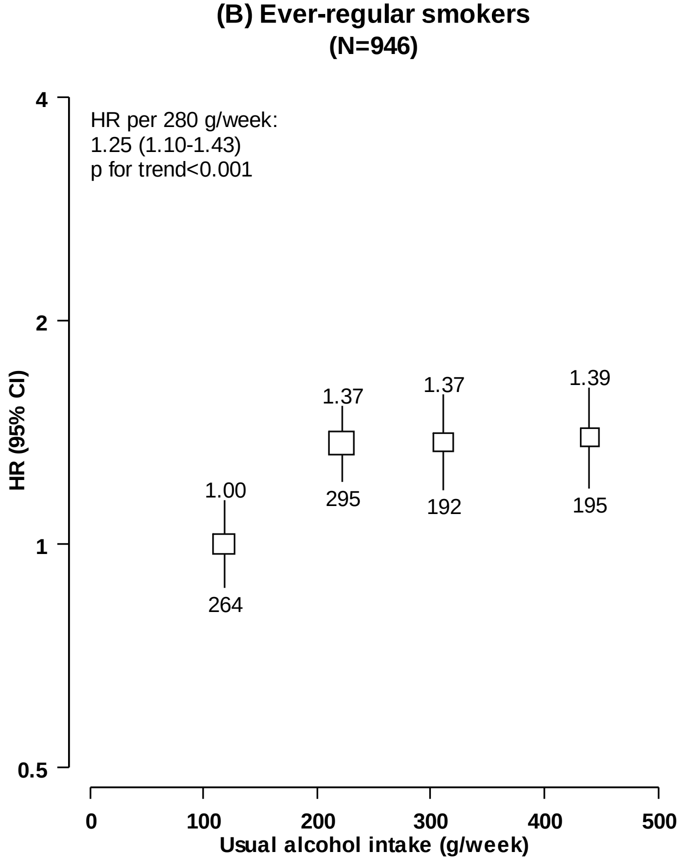

P for heterogeneity = 0.58. Conventions are as in Figure S1.

Figure S5. Associations of alcohol consumption with other site-specific cancers, in male current regular drinkers

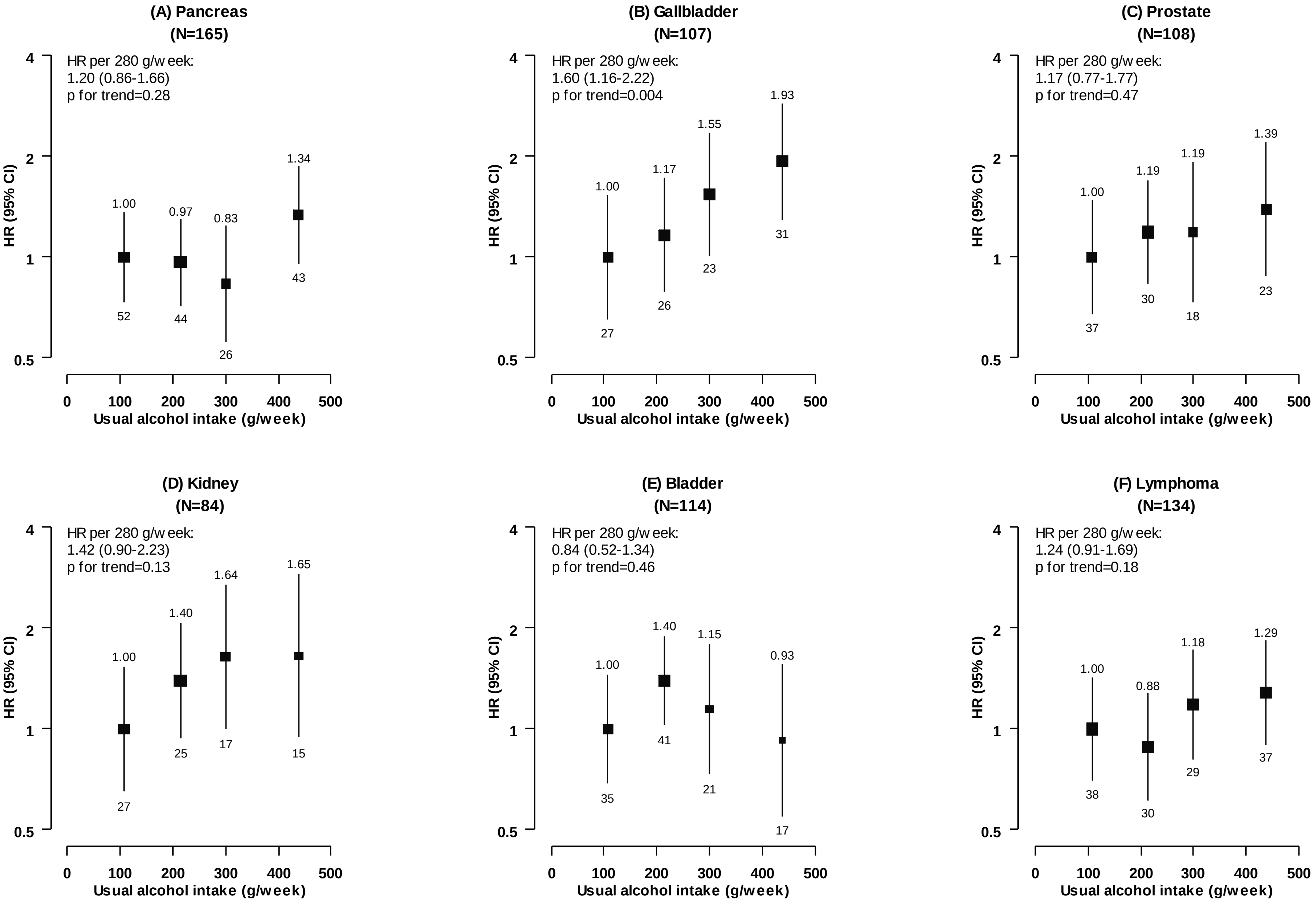

Conventions are as in Figure S1.

Figure S6. Associations of alcohol with other less common site-specific cancers, in male current regular drinkers

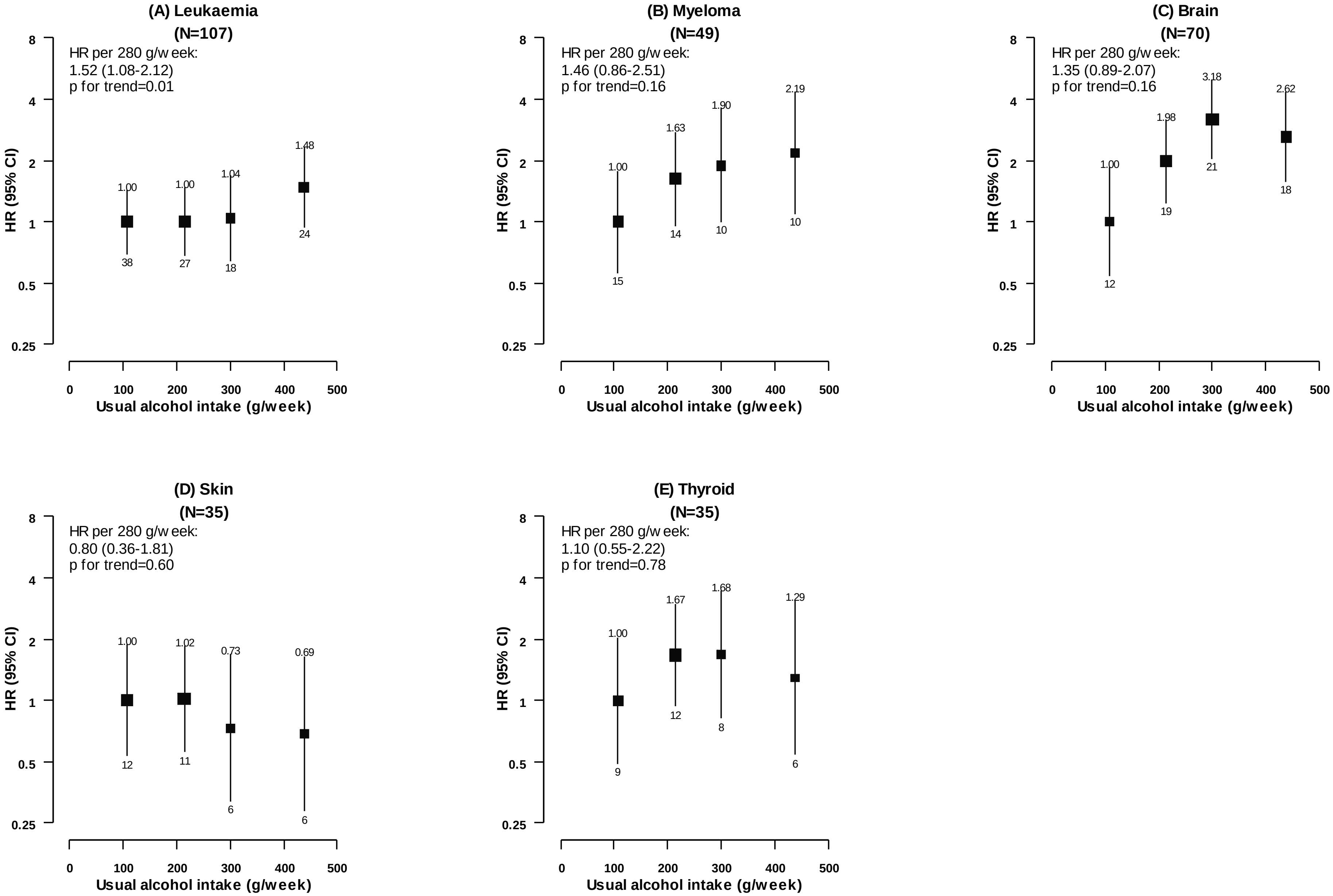

Conventions are as in Figure S1.

Figure S7. Associations of alcohol consumption with total cancer, by smoking status in male current regular drinkers

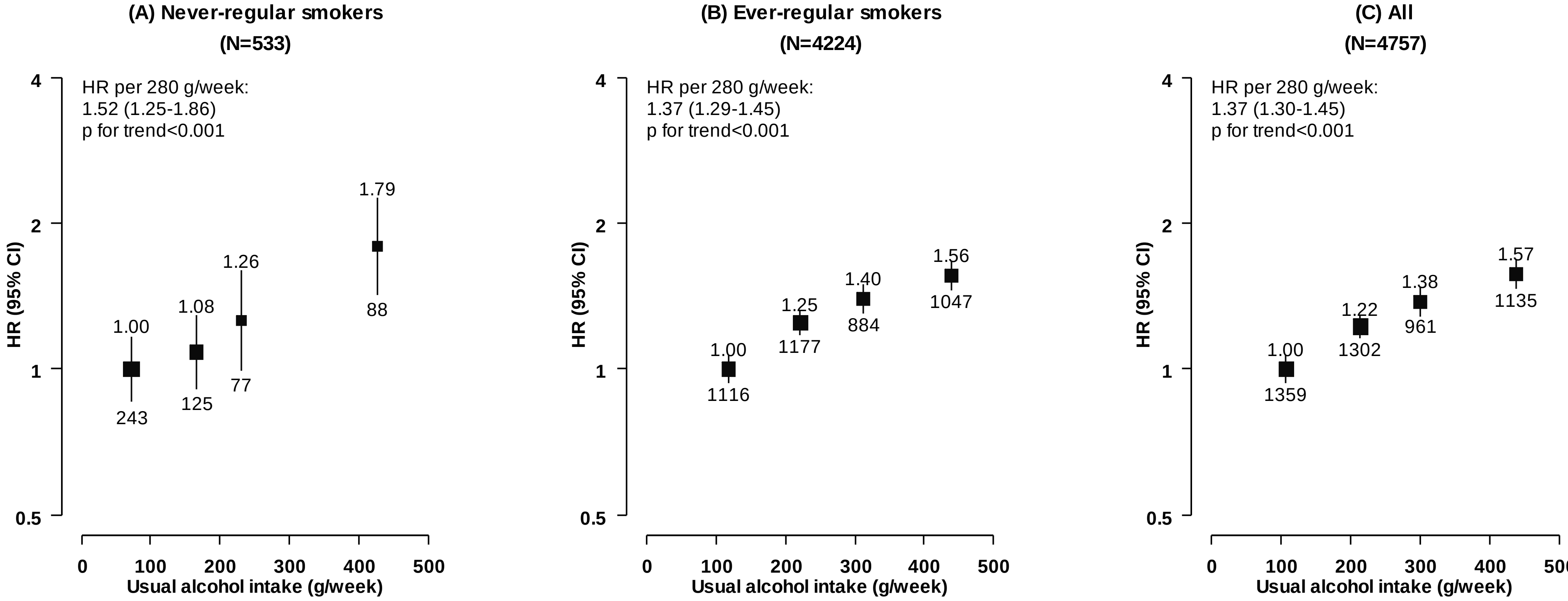

Conventions are as in Figure S1.

Figure S8. HRs of oesophageal cancer, IARC alcohol-related cancers, other cancers, and total cancer associated with alcohol intake among male current regular drinkers over the range of 0-2000 g/week

(B) Oesophageal cancer

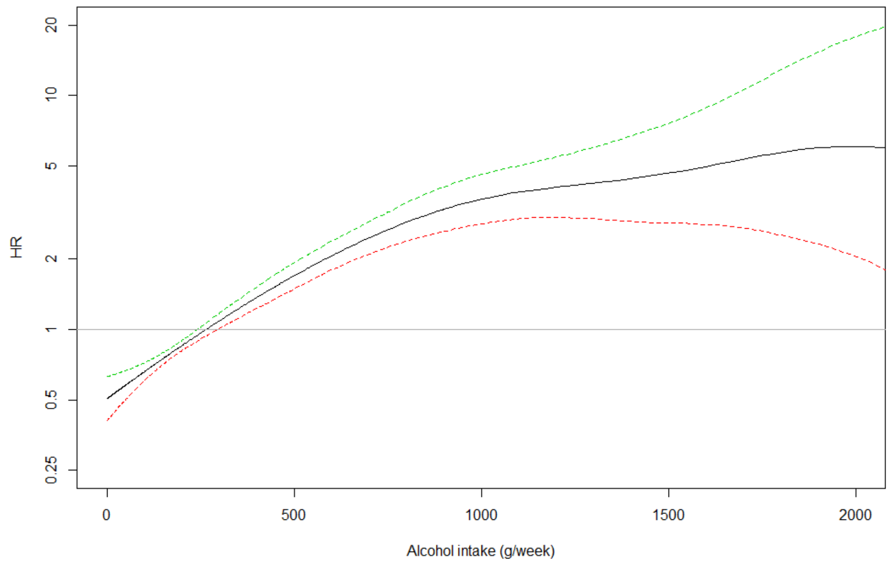

(A) IARC alcohol-related cancers

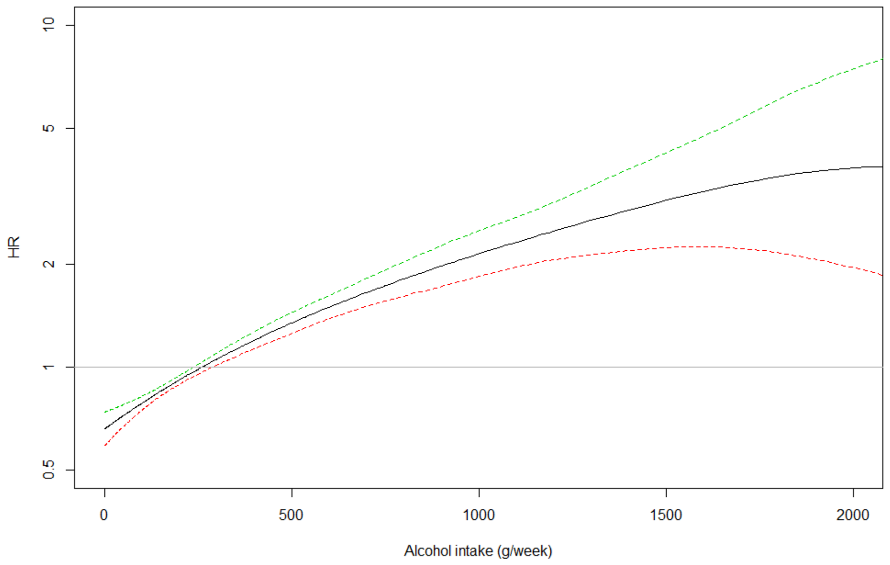

(C) Other cancers of known sites

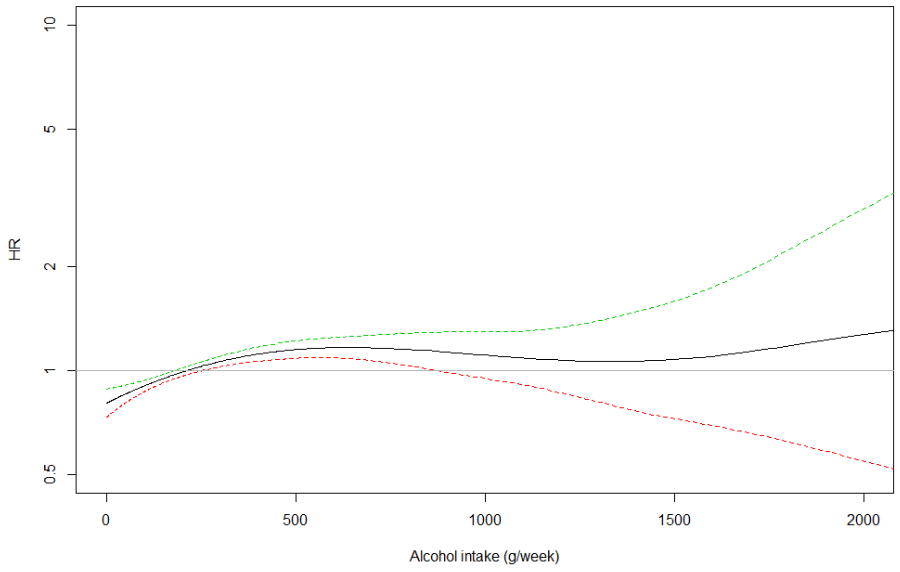

(D) All cancers

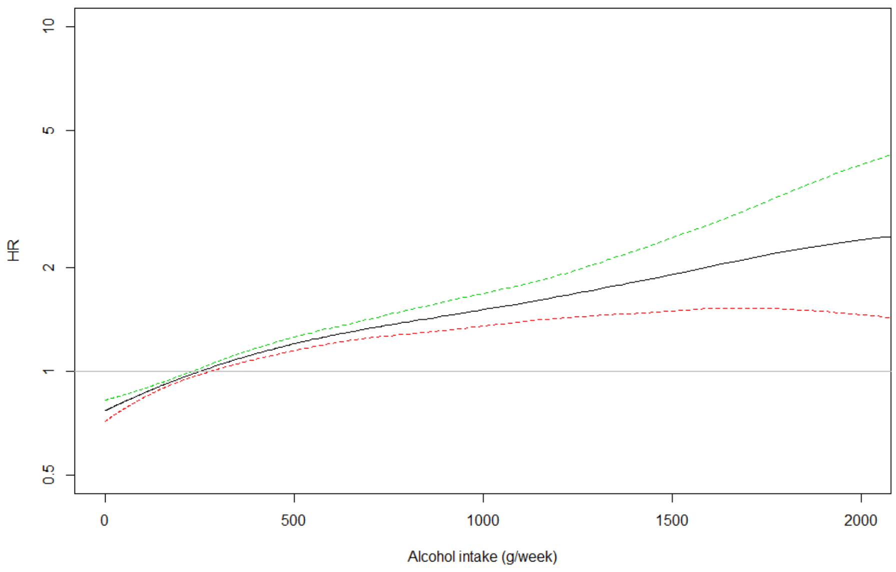

Figure S9. Adjusted HRs per 280 g/week higher usual alcohol intake for IARC alcohol-related cancers and total cancer, by population subgroups in male current regular drinkers

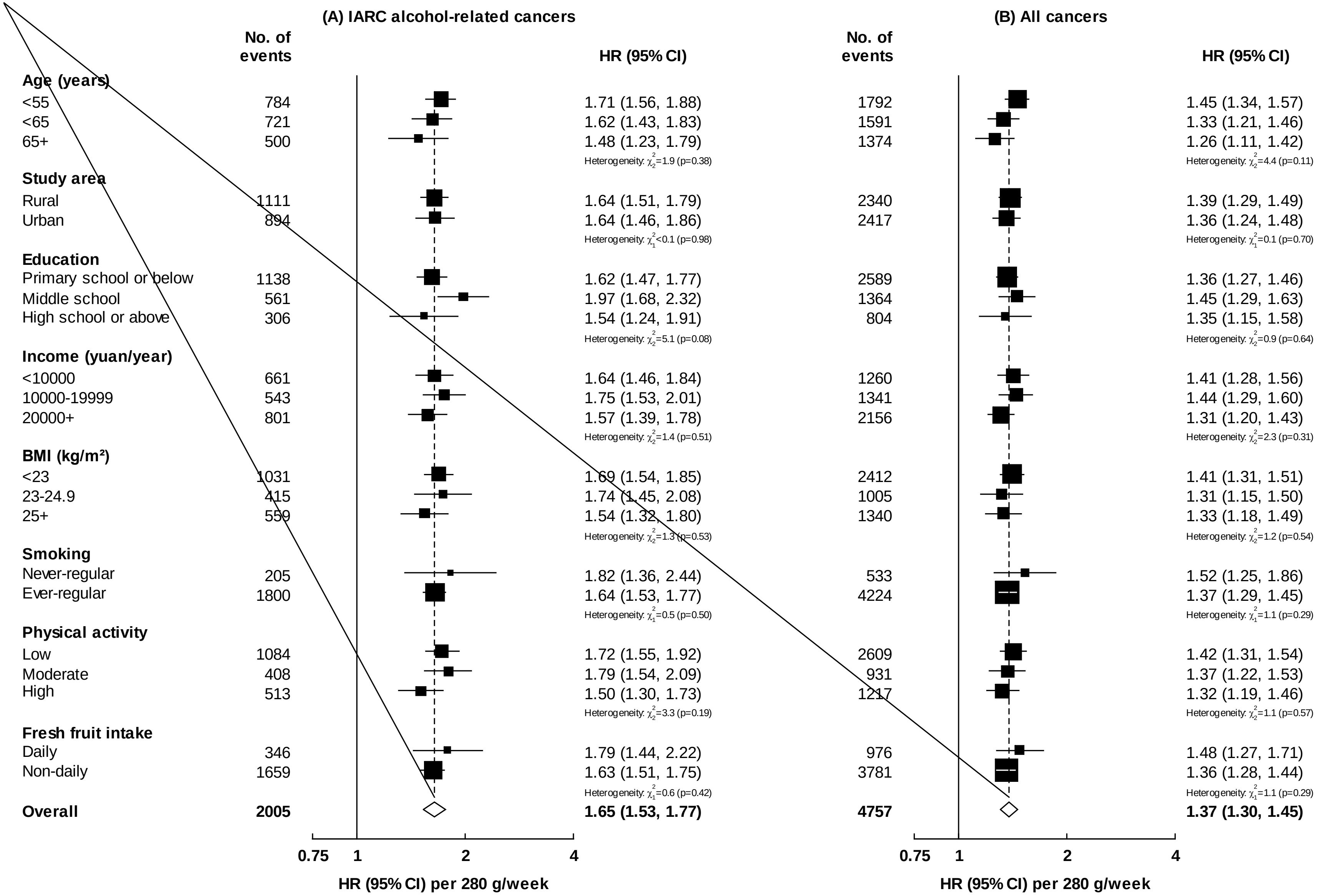

The analysis assumed a linear association of per 280 g/week higher usual alcohol intake. Conventions are as in Figure S1.

Figure S10. Adjusted HRs per 280 g/week higher usual alcohol intake for common cancers, by hepatitis B infection status in male current regular drinkers

	No. of events	HR (95% CI) per 280 g/week
**Oesophagus**		
HBsAg-	618	1.96 (1.77, 2.17)
HBsAg+	12	1.78 (0.45, 7.11)
		Heterogeneity: χ^2_1<0.1 (p=0.89)
**Colon and rectum**		
HBsAg-	540	1.19 (0.99, 1.44)
HBsAg+	24	1.14 (0.45, 2.88)
		Heterogeneity: χ^2_1<0.1 (p=0.92)
**Liver**		
HBsAg-	413	1.37 (1.14, 1.65)
HBsAg+	134	1.75 (1.22, 2.51)
		Heterogeneity: χ^2_1=1.4 (p=0.24)
**Stomach**		
HBsAg-	708	1.11 (0.94, 1.31)
HBsAg+	23	0.97 (0.36, 2.60)
		Heterogeneity: χ^2_1=0.1 (p=0.79)
**Lung**		
HBsAg-	972	1.24 (1.09, 1.41)
HBsAg+	23	1.95 (0.69, 5.48)
		Heterogeneity: χ^2_1=0.7 (p=0.40)
**IARC alcohol-related cancers**		
HBsAg-	1744	1.63 (1.51, 1.76)
HBsAg+	190	1.41 (1.04, 1.91)
		Heterogeneity: χ^2_1=0.8 (p=0.36)
**Other cancers of known sites**		
HBsAg-	2875	1.17 (1.08, 1.26)
HBsAg+	87	1.31 (0.81, 2.11)
		Heterogeneity: χ^2_1=0.2 (p=0.63)
**All cancers**		
HBsAg-	4353	1.36 (1.28, 1.44)
HBsAg+	257	1.35 (1.04, 1.76)
		Heterogeneity: χ^2_1<0.1 (p=0.99)

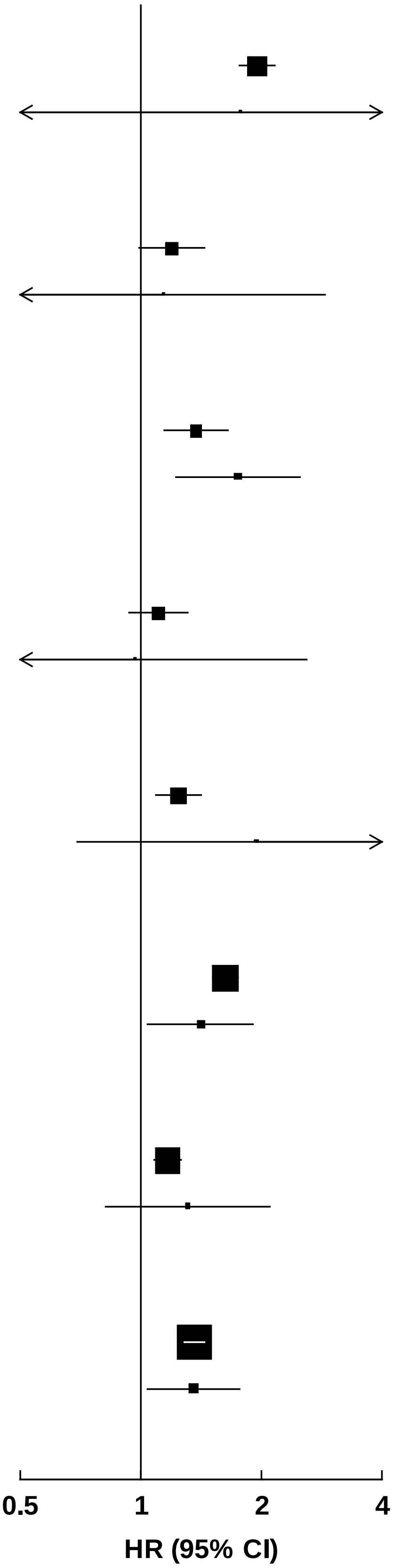

The analysis assumed a linear association of per 280 g/week higher usual alcohol intake. HBsAg, hepatitis B surface antigen. Conventions are as in Figure S1.

Table S10. Adjusted HRs for common cancers associated with daily drinking, by total weekly amount in male current regular drinkers

Cancer site		All current regular N	1-5 days/week N	1-5 days/week HR (95% CI)	6-7 days/week N	6-7 days/week Full model[a] HR (95% CI)	Full model[a] *P*	Further adjusted for alcohol consumption HR (95% CI)	Further adjusted for alcohol consumption *P*
Mouth and throat	<280 g/week	106	36	1.00 (Reference)	70	1.48 (0.97-2.26)	0.07	1.06 (0.63-1.76)	0.83
	≥280 g/week	130	5	1.00 (Reference)	125	1.40 (0.56-3.53)	0.47	1.18 (0.47-2.99)	0.73
Oesophagus	<280 g/week	247	86	1.00 (Reference)	161	1.77 (1.33-2.34)	<0.001	1.51 (1.08-2.12)	0.02
	≥280 g/week	408	15	1.00 (Reference)	393	1.72 (1.01-2.95)	0.047	1.47 (0.86-2.52)	0.16
Colon and rectum	<280 g/week	349	131	1.00 (Reference)	218	1.33 (1.05-1.68)	0.02	1.26 (0.95-1.66)	0.11
	≥280 g/week	226	13	1.00 (Reference)	213	0.99 (0.54-1.83)	0.98	0.99 (0.53-1.83)	0.96
Liver	<280 g/week	300	122	1.00 (Reference)	178	1.28 (1.00-1.65)	0.051	1.23 (0.91-1.65)	0.19
	≥280 g/week	273	14	1.00 (Reference)	259	1.30 (0.74-2.30)	0.37	1.19 (0.67-2.12)	0.56
Stomach	<280 g/week	421	166	1.00 (Reference)	255	1.12 (0.91-1.38)	0.29	1.12 (0.87-1.44)	0.37
	≥280 g/week	336	13	1.00 (Reference)	323	1.15 (0.65-2.06)	0.63	1.20 (0.67-2.15)	0.54
Lung	<280 g/week	608	224	1.00 (Reference)	384	1.18 (0.99-1.41)	0.06	1.04 (0.84-1.28)	0.74
	≥280 g/week	409	20	1.00 (Reference)	389	1.13 (0.70-1.81)	0.61	1.08 (0.67-1.74)	0.74
IARC alcohol-related cancers	<280 g/week	993	370	1.00 (Reference)	623	1.44 (1.26-1.66)	<0.001	1.32 (1.12-1.56)	0.001
	≥280 g/week	1012	49	1.00 (Reference)	963	1.29 (0.96-1.75)	0.10	1.15 (0.85-1.56)	0.37
Other cancers of known sites (non-IARC alcohol-related)	<280 g/week	1796	689	1.00 (Reference)	1107	1.16 (1.05-1.29)	0.004	1.06 (0.94-1.20)	0.34
	≥280 g/week	1250	57	1.00 (Reference)	1193	1.16 (0.88-1.53)	0.30	1.16 (0.88-1.54)	0.30
All cancers[b]	<280 g/week	2661	1027	1.00 (Reference)	1634	1.23 (1.13-1.34)	<0.001	1.14 (1.03-1.26)	0.01
	≥280 g/week	2096	103	1.00 (Reference)	1993	1.16 (0.94-1.43)	0.16	1.09 (0.88-1.35)	0.42

HR, hazard ratio; CI, confidence interval; IARC, International Agency for Research on Cancer.

Participants with self-reported prior cancer were excluded from all analyses. Participants with self-reported prior chronic hepatitis/liver cirrhosis were further excluded from analysis of liver cancer, and participants with self-reported prior tuberculosis, emphysema/bronchitis, or chronic obstructive pulmonary disease were further excluded from analysis of lung cancer.

[a] Cox models are stratified by age-at-risk and study area, and adjusted for education, income, smoking, physical activity, fruit intake, body mass index, and family history of cancer.

[b] All cancers included ill-defined neoplasm and are patient-based.

Table S11. Adjusted HRs for common cancers associated with heavy episodic drinking, by total weekly amount in male current regular drinkers

			<60 g/session		60+ g/session (i.e. HED)				
						Full model[a]		Further adjusted for alcohol consumption	
Cancer site		All current regular N	N	HR (95% CI)	N	HR (95% CI)	*P*	HR (95% CI)	*P*
Mouth and throat	<280 g/week	106	96	1.00 (Reference)	10	1.31 (0.66-2.60)	0.45	1.23 (0.62-2.45)	0.56
	≥280 g/week	130	22	1.00 (Reference)	108	1.06 (0.63-1.80)	0.82	0.81 (0.47-1.40)	0.45
Oesophagus	<280 g/week	247	231	1.00 (Reference)	16	0.81 (0.48-1.37)	0.43	0.75 (0.44-1.27)	0.28
	≥280 g/week	408	50	1.00 (Reference)	358	1.33 (0.95-1.86)	0.09	1.02 (0.72-1.44)	0.91
Colon and rectum	<280 g/week	349	324	1.00 (Reference)	25	0.93 (0.61-1.44)	0.75	0.90 (0.59-1.39)	0.65
	≥280 g/week	226	59	1.00 (Reference)	167	0.87 (0.62-1.22)	0.43	0.83 (0.56-1.21)	0.33
Liver	<280 g/week	300	273	1.00 (Reference)	27	1.02 (0.67-1.56)	0.93	0.99 (0.64-1.52)	0.96
	≥280 g/week	273	56	1.00 (Reference)	217	1.15 (0.82-1.60)	0.42	0.96 (0.67-1.38)	0.82
Stomach	<280 g/week	421	393	1.00 (Reference)	28	0.97 (0.65-1.46)	0.90	0.97 (0.64-1.45)	0.87
	≥280 g/week	336	102	1.00 (Reference)	234	0.80 (0.62-1.05)	0.11	0.82 (0.61-1.12)	0.21
Lung	<280 g/week	608	562	1.00 (Reference)	46	1.20 (0.87-1.65)	0.26	1.17 (0.85-1.61)	0.33
	≥280 g/week	409	97	1.00 (Reference)	312	1.01 (0.78-1.31)	0.93	0.92 (0.69-1.22)	0.55
IARC alcohol-related cancers	<280 g/week	993	917	1.00 (Reference)	76	0.94 (0.73-1.21)	0.63	0.90 (0.70-1.15)	0.40
	≥280 g/week	1012	181	1.00 (Reference)	831	1.12 (0.93-1.34)	0.24	0.90 (0.74-1.09)	0.27
Other cancers of known sites (non-IARC alcohol-related)	<280 g/week	1796	1675	1.00 (Reference)	121	1.01 (0.83-1.23)	0.93	0.98 (0.81-1.20)	0.87
	≥280 g/week	1250	322	1.00 (Reference)	928	0.92 (0.80-1.07)	0.28	0.90 (0.77-1.06)	0.21
All cancers[b]	<280 g/week	2661	2471	1.00 (Reference)	190	0.98 (0.84-1.14)	0.78	0.95 (0.81-1.11)	0.52
	≥280 g/week	2096	461	1.00 (Reference)	1635	1.01 (0.90-1.14)	0.85	0.88 (0.77-1.00)	0.050

HR, hazard ratio; CI, confidence interval; HED, heavy episodic drinking; IARC, International Agency for Research on Cancer.

Participants with self-reported prior cancer were excluded from all analyses. Participants with self-reported prior chronic hepatitis/liver cirrhosis were further excluded from analysis of liver cancer, and participants with self-reported prior tuberculosis, emphysema/bronchitis, or chronic obstructive pulmonary disease were further excluded from analysis of lung cancer.

[a] Cox models are stratified by age-at-risk and study area, and adjusted for education, income, smoking, physical activity, fruit intake, body mass index, and family history of cancer.

[b] All cancers included ill-defined neoplasm and are patient-based.

Figure S11. Adjusted HRs for common cancers associated with duration of regular drinking, in male current regular drinkers

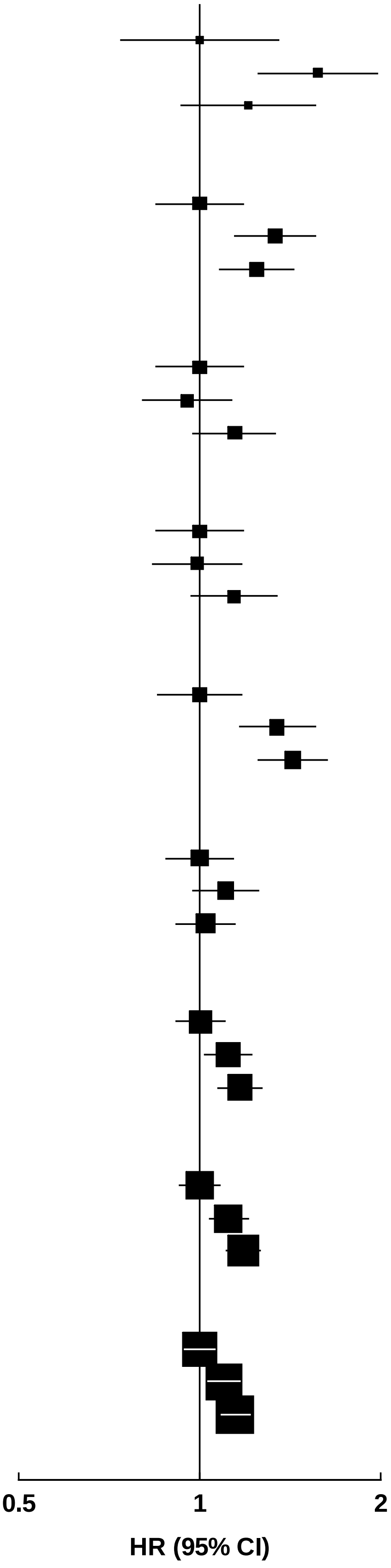

	No. of events	HR (95%CI)
**Mouth and throat**		
<20 years	51	1.00 (0.74, 1.35)
20-29 years	82	1.57 (1.25, 1.96)
30+ years	103	1.20 (0.93, 1.56)
		Heterogeneity: χ^2_2=6.1 (p=0.047)
**Oesophagus**		
<20 years	155	1.00 (0.84, 1.18)
20-29 years	181	1.33 (1.14, 1.55)
30+ years	319	1.24 (1.08, 1.43)
		Heterogeneity: χ^2_2=6.8 (p=0.03)
**Colon and rectum**		
<20 years	165	1.00 (0.85, 1.18)
20-29 years	138	0.95 (0.80, 1.13)
30+ years	272	1.14 (0.98, 1.34)
		Heterogeneity: χ^2_2=2.7 (p=0.26)
**Liver**		
<20 years	165	1.00 (0.85, 1.18)
20-29 years	143	0.99 (0.84, 1.18)
30+ years	265	1.14 (0.97, 1.34)
		Heterogeneity: χ^2_2=1.8 (p=0.40)
**Stomach**		
<20 years	169	1.00 (0.85, 1.18)
20-29 years	202	1.34 (1.17, 1.55)
30+ years	386	1.43 (1.25, 1.63)
		Heterogeneity: χ^2_2=12.4 (p=0.002)
**Lung**		
<20 years	263	1.00 (0.88, 1.14)
20-29 years	275	1.10 (0.98, 1.25)
30+ years	479	1.02 (0.91, 1.14)
		Heterogeneity: χ^2_2=1.4 (p=0.49)
**IARC alcohol-related cancers**		
<20 years	529	1.00 (0.91, 1.10)
20-29 years	533	1.12 (1.02, 1.22)
30+ years	943	1.17 (1.07, 1.27)
		Heterogeneity: χ^2_2=6.2 (p=0.045)
**Other cancers of known sites**		
<20 years	785	1.00 (0.93, 1.08)
20-29 years	770	1.12 (1.04, 1.20)
30+ years	1491	1.18 (1.11, 1.26)
		Heterogeneity: χ^2_2=11.1 (p=0.004)
**All cancers**		
<20 years	1258	1.00 (0.94, 1.06)
20-29 years	1234	1.10 (1.04, 1.16)
30+ years	2265	1.14 (1.08, 1.21)
		Heterogeneity: χ^2_2=11.4 (p=0.003)

Cox models are stratified by age-at-risk and study area, and adjusted for education, income, smoking status, physical activity, fresh fruit intake, body mass index, family history of cancer, total weekly intake, and baseline age. Conventions are as in Figure S1.

Figure S12. Adjusted HRs per 280 g/week higher usual alcohol intake for common cancers, by flushing status in male current regular drinkers

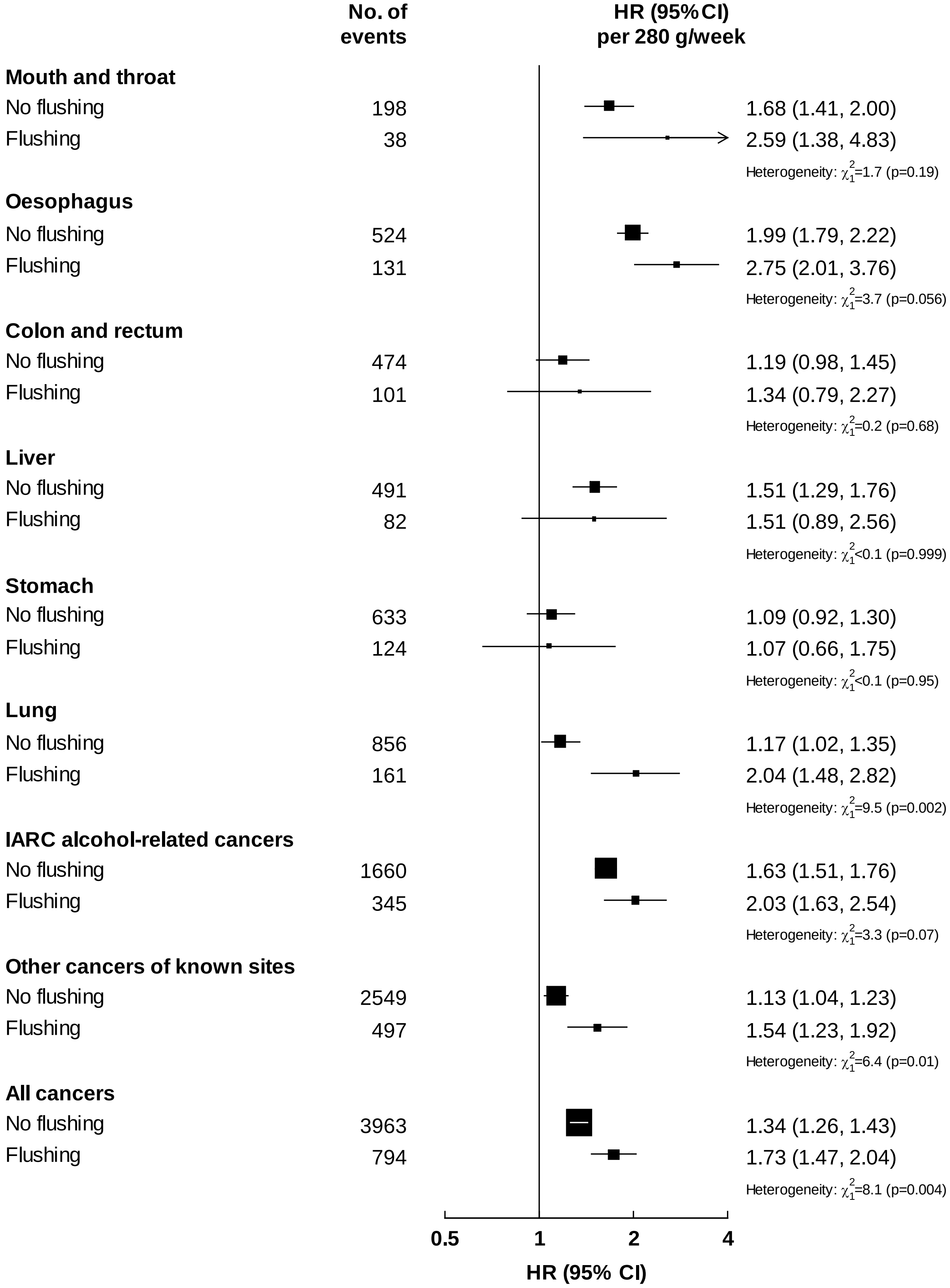

The analysis assumed a linear association of per 280 g/week higher usual alcohol intake. Conventions are as in Figure S1.

Table S12. Adjusted HRs per 280 g/week higher usual alcohol intake for incident cancers, in male current regular drinkers – in sequentially adjusted models

	Mouth and throat HR (95% CI)	Oesophagus HR (95% CI)	Colon and rectum HR (95% CI)	Liver HR (95% CI)	Stomach HR (95% CI)	Pancreas HR (95% CI)	Lung HR (95% CI)	Gallbladder HR (95% CI)	IARC alcohol-related cancers HR (95% CI)	Other cancers of known sites HR (95% CI)	All cancers[a] HR (95% CI)
**Stratified by age-at-risk & study area**	1.80 (1.56-2.08)	2.11 (1.93-2.31)	1.26 (1.06-1.49)	1.57 (1.37-1.81)	1.18 (1.01-1.37)	1.19 (0.87-1.63)	1.42 (1.27-1.60)	1.63 (1.19-2.23)	1.74 (1.63-1.85)	1.26 (1.17-1.35)	1.46 (1.39-1.54)
**Further adjusted for**											
+ Education	1.80 (1.55-2.09)	2.08 (1.90-2.27)	1.25 (1.05-1.49)	1.57 (1.36-1.81)	1.14 (0.98-1.33)	1.19 (0.86-1.64)	1.40 (1.25-1.58)	1.66 (1.21-2.27)	1.72 (1.61-1.84)	1.24 (1.15-1.33)	1.45 (1.37-1.52)
+ Income	1.80 (1.55-2.09)	2.06 (1.89-2.26)	1.25 (1.05-1.49)	1.57 (1.36-1.80)	1.14 (0.98-1.33)	1.20 (0.87-1.64)	1.40 (1.24-1.58)	1.65 (1.20-2.25)	1.72 (1.61-1.84)	1.24 (1.15-1.33)	1.45 (1.37-1.52)
+ Smoking	1.73 (1.48-2.03)	1.96 (1.78-2.15)	1.22 (1.02-1.45)	1.53 (1.32-1.77)	1.11 (0.95-1.30)	1.20 (0.87-1.66)	1.26 (1.11-1.43)	1.62 (1.18-2.23)	1.66 (1.55-1.78)	1.18 (1.09-1.27)	1.38 (1.31-1.46)
+ Physical activity	1.75 (1.49-2.06)	1.97 (1.79-2.17)	1.22 (1.02-1.45)	1.53 (1.32-1.77)	1.11 (0.95-1.30)	1.20 (0.87-1.66)	1.26 (1.11-1.43)	1.63 (1.18-2.25)	1.66 (1.55-1.78)	1.18 (1.09-1.27)	1.38 (1.31-1.46)
+ Fruit intake	1.74 (1.48-2.05)	1.96 (1.78-2.16)	1.20 (1.01-1.44)	1.52 (1.31-1.77)	1.11 (0.94-1.30)	1.20 (0.87-1.66)	1.26 (1.11-1.43)	1.61 (1.16-2.22)	1.65 (1.54-1.77)	1.18 (1.09-1.27)	1.38 (1.30-1.45)
+ BMI	1.74 (1.48-2.05)	2.00 (1.81-2.21)	1.20 (1.00-1.43)	1.52 (1.31-1.77)	1.11 (0.94-1.30)	1.20 (0.86-1.66)	1.25 (1.10-1.42)	1.60 (1.16-2.22)	1.65 (1.54-1.77)	1.17 (1.09-1.27)	1.38 (1.30-1.45)
**+ Family history of cancer (Main Model)**	**1.74 (1.48-2.05)**	**1.98 (1.79-2.18)**	**1.19 (1.00-1.43)**	**1.52 (1.31-1.76)**	**1.11 (0.94-1.30)**	**1.20 (0.86-1.66)**	**1.25 (1.10-1.42)**	**1.60 (1.16-2.22)**	**1.65 (1.53-1.77)**	**1.17 (1.09-1.27)**	**1.37 (1.30-1.45)**
**+** Self-reported health status	1.75 (1.49-2.07)	1.97 (1.79-2.18)	1.19 (1.00-1.43)	1.52 (1.32-1.77)	1.10 (0.94-1.29)	1.20 (0.87-1.67)	1.26 (1.11-1.43)	1.62 (1.17-2.23)	1.65 (1.54-1.77)	1.18 (1.09-1.27)	1.38 (1.30-1.45)
+ Meat intake	1.76 (1.49-2.08)	1.97 (1.79-2.18)	1.20 (1.00-1.43)	1.52 (1.31-1.76)	1.11 (0.94-1.30)	1.20 (0.86-1.66)	1.26 (1.11-1.43)	1.61 (1.17-2.23)	1.65 (1.53-1.77)	1.18 (1.09-1.27)	1.38 (1.30-1.46)
+ Preserved vegetable intake	1.75 (1.48-2.07)	1.99 (1.79-2.20)	1.20 (1.00-1.43)	1.52 (1.31-1.76)	1.10 (0.94-1.30)	1.20 (0.86-1.66)	1.26 (1.11-1.43)	1.62 (1.18-2.24)	1.64 (1.53-1.76)	1.18 (1.09-1.27)	1.38 (1.30-1.45)

HR, hazard ratio; CI, confidence interval; IARC, International Agency for Research on Cancer; BMI, body mass index.

Participants with prior cancer were excluded from all analyses. Participants with prior chronic hepatitis/cirrhosis were further excluded from the analysis of liver cancer, and participants with prior tuberculosis, emphysema/bronchitis, or chronic obstructive pulmonary disease were further excluded from the analysis of lung cancer.

[a] All cancers included ill-defined neoplasm and are patient-based.

Figure S13. Associations of alcohol consumption with IARC alcohol-related cancers, other cancers, and total cancer, in all men

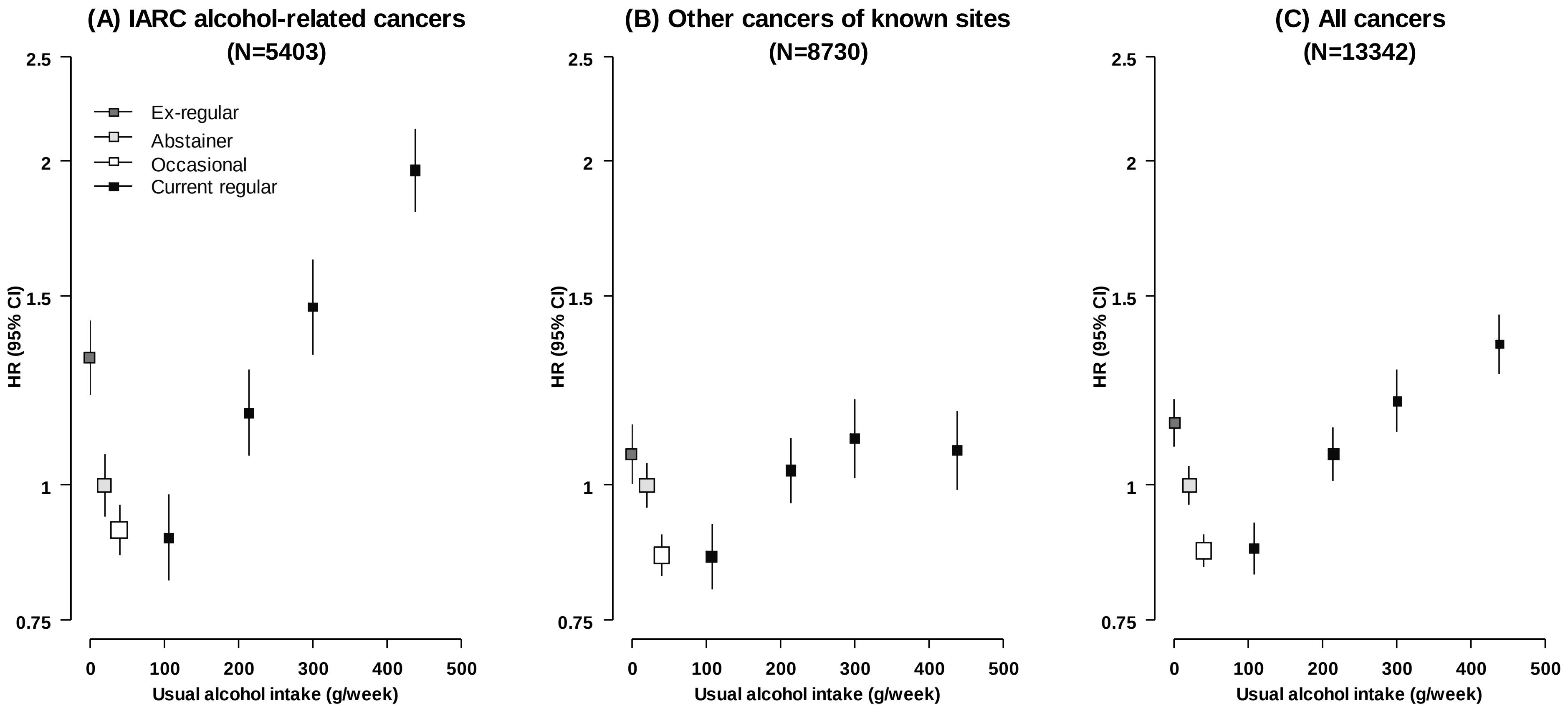

Conventions are as in Figure S1.

Figure S14. Associations of alcohol consumption with common cancers in all men

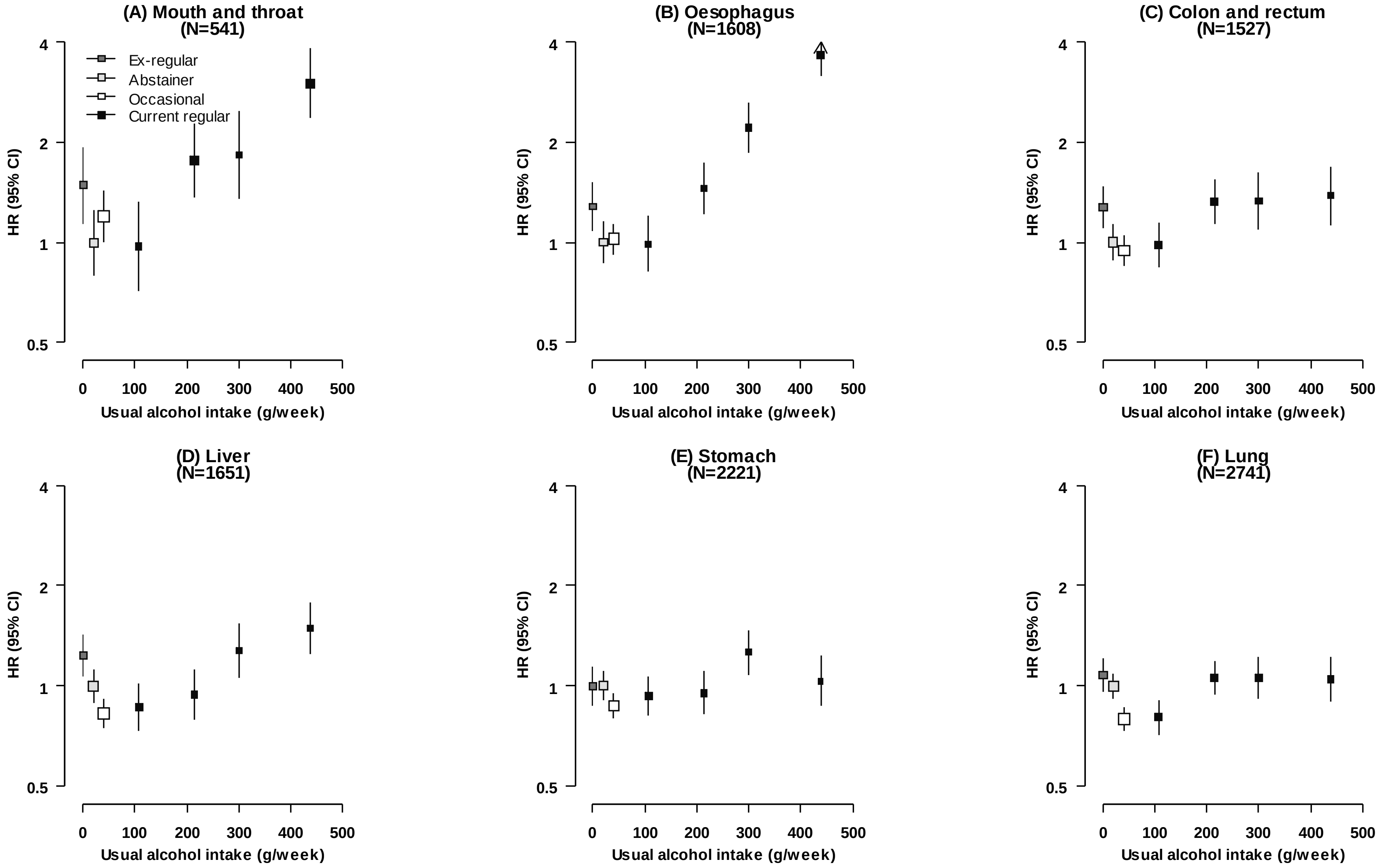

Conventions are as in Figure S1.

Figure S15. Associations of alcohol consumption with other site-specific cancers, in all men

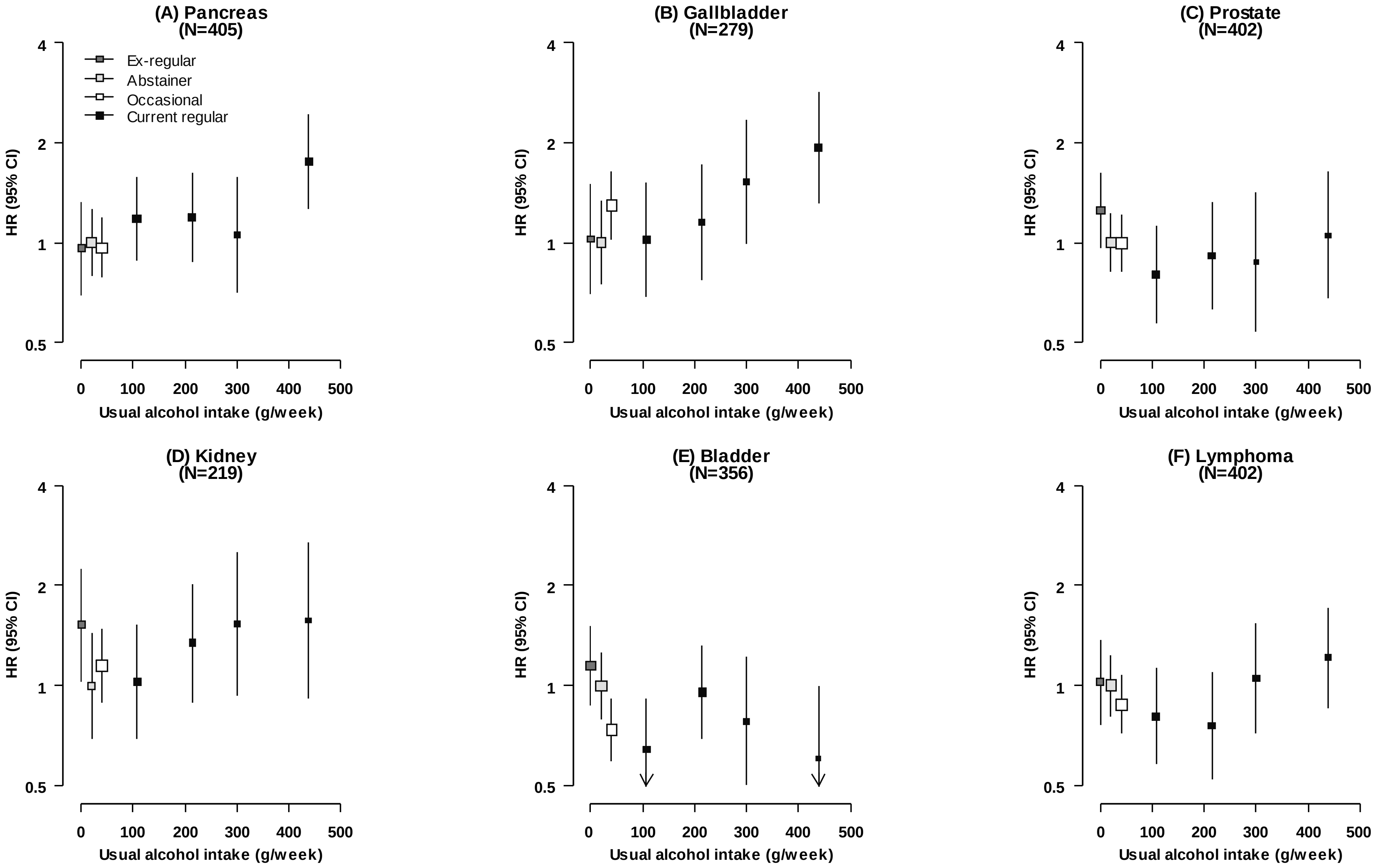

Conventions are as in Figure S1.

Figure S16. Associations of alcohol consumption with other less common site-specific cancers, in all men

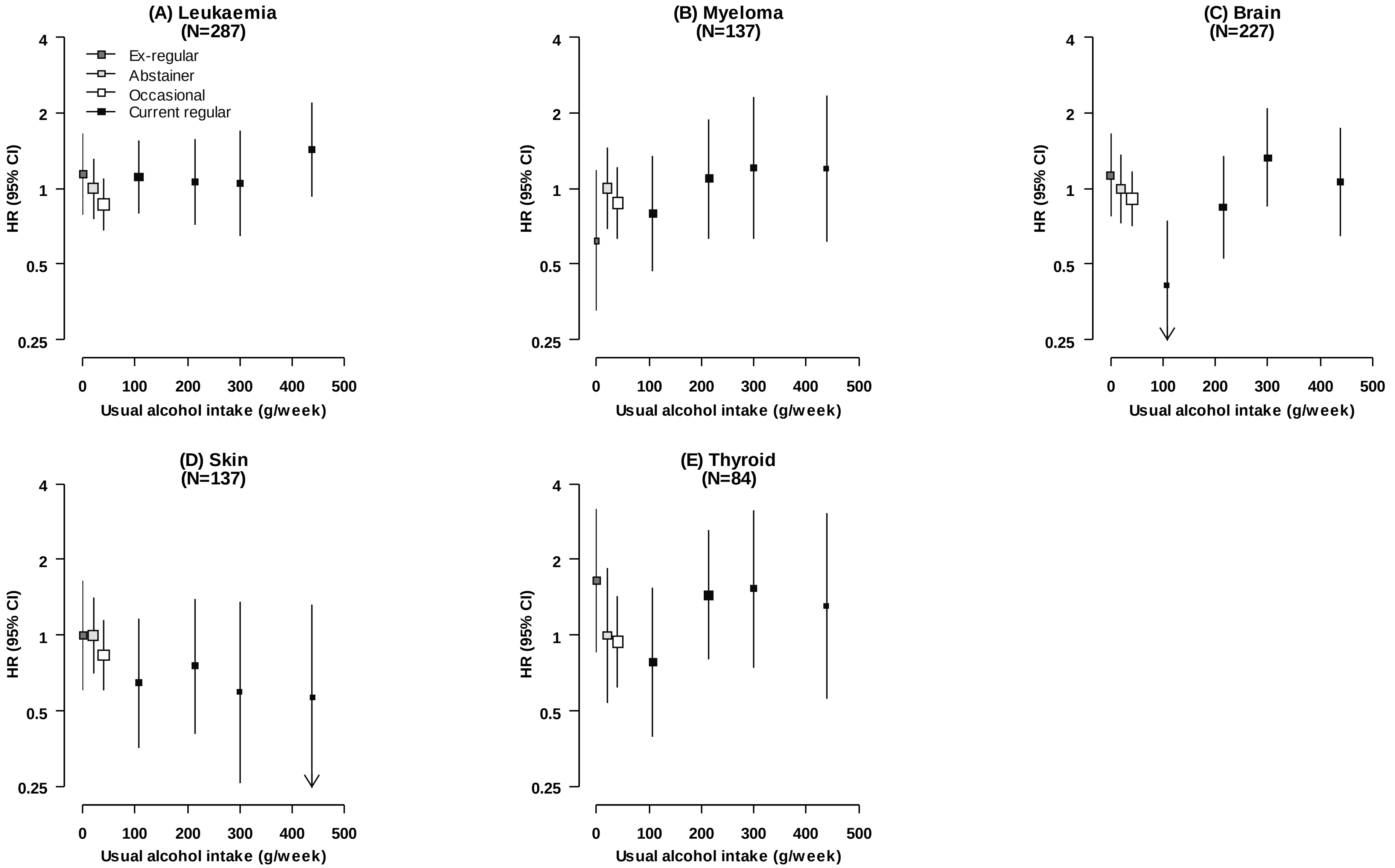

Conventions are as in Figure S1.

Table S13. Adjusted HRs for incident cancers associated with alcohol drinking status, after various exclusions of participants in men

	All men	Abstainers		Ex-regular drinkers		Occasional drinkers		Current regular drinkers		
	N	N	HR (95% CI)	N	HR (95% CI)	N	HR (95% CI)	N	HR (95% CI)	*P* [a]
**Excluding first three years of follow-up**										
IARC alcohol-related cancers	3977	735	1.00 (0.93-1.08)	464	1.27 (1.16-1.40)	1252	0.97 (0.92-1.03)	1526	1.33 (1.26-1.41)	<0.001
Other cancers of known sites (Non-IARC alcohol-related)	6642	1519	1.00 (0.95-1.06)	767	1.04 (0.97-1.12)	1966	0.90 (0.86-0.94)	2390	1.04 (0.99-1.08)	0.32
All cancers[b]	9952	2137	1.00 (0.96-1.05)	1155	1.11 (1.05-1.18)	3008	0.92 (0.88-0.95)	3652	1.12 (1.08-1.16)	<0.001
**Excluding participants with poor self-reported health status at baseline**										
IARC alcohol-related cancers	4780	900	1.00 (0.93-1.07)	524	1.28 (1.18-1.40)	1509	0.95 (0.90-1.00)	1847	1.29 (1.23-1.36)	<0.001
Other cancers of known sites (Non-IARC alcohol-related)	7809	1819	1.00 (0.95-1.05)	863	1.05 (0.98-1.13)	2295	0.87 (0.84-0.91)	2832	1.00 (0.96-1.04)	0.9997
All cancers[b]	11873	2588	1.00 (0.96-1.04)	1298	1.12 (1.06-1.18)	3585	0.89 (0.86-0.92)	4402	1.09 (1.05-1.12)	0.002
**Excluding participants with self-reported prior chronic disease[c] at baseline**										
IARC alcohol-related cancers	3815	668	1.00 (0.92-1.08)	342	1.25 (1.12-1.39)	1240	1.00 (0.94-1.06)	1565	1.40 (1.33-1.48)	<0.001
Other cancers of known sites (Non-IARC alcohol-related)	6188	1408	1.00 (0.95-1.06)	597	1.08 (0.99-1.17)	1855	0.88 (0.84-0.93)	2328	1.01 (0.97-1.06)	0.77
All cancers[b]	9423	1979	1.00 (0.95-1.05)	892	1.14 (1.06-1.21)	2900	0.91 (0.88-0.95)	3652	1.12 (1.08-1.16)	<0.001
**Excluding all of above**										
IARC alcohol-related cancers	2676	443	1.00 (0.91-1.10)	218	1.24 (1.08-1.41)	886	1.05 (0.98-1.13)	1129	1.44 (1.35-1.53)	<0.001
Other cancers of known sites (Non-IARC alcohol-related)	4443	969	1.00 (0.94-1.07)	374	1.01 (0.91-1.12)	1372	0.92 (0.87-0.97)	1728	1.04 (0.99-1.10)	0.31
All cancers[b]	6655	1342	1.00 (0.94-1.06)	564	1.10 (1.01-1.19)	2096	0.95 (0.91-0.99)	2653	1.15 (1.10-1.20)	<0.001

HR, hazard ratio; CI, confidence interval; IARC, International Agency for Research on Cancer.

Cox models are stratified by age-at-risk and study area, and adjusted for education, income, smoking, physical activity, fruit intake, body mass index, and family history of cancer.

Participants with self-reported prior cancer were excluded from all analyses.

[a] *P* for association comparing current regular drinkers vs. abstainers.

[b] All cancers included ill-defined neoplasm and are patient-based.

[c] Chronic diseases included self-reported coronary heart disease, stroke, transient ischaemic attack, diabetes, tuberculosis, chronic hepatitis/liver cirrhosis, rheumatoid arthritis, peptic ulcer, emphysema/bronchitis, gallstone/gallbladder disease, and kidney disease.

Table S14. Adjusted HRs for incident cancers associated with level of alcohol consumption, after various exclusions of participants in male current regular drinkers

	All current regular	<140 g/week		140-279 g/week		280-419 g/week		420+ g/week		Per 280 g/week increase		
	N	N	HR (95% CI)	N	HR (95% CI)	N	HR (95% CI)	N	HR (95% CI)	N	HR (95% CI)	P_{trend}
**Excluding first three years of follow-up**												
IARC alcohol-related cancers	1526	372	1.00 (0.90-1.12)	384	1.31 (1.18-1.44)	317	1.61 (1.44-1.80)	453	2.15 (1.94-2.39)	1526	1.66 (1.53-1.80)	<0.001
Other cancers of known sites (Non-IARC alcohol-related)	2390	714	1.00 (0.92-1.08)	682	1.22 (1.13-1.31)	498	1.34 (1.23-1.46)	496	1.32 (1.20-1.45)	2390	1.19 (1.09-1.30)	<0.001
All cancers[a]	3652	1041	1.00 (0.94-1.07)	994	1.22 (1.15-1.30)	745	1.38 (1.29-1.49)	872	1.57 (1.46-1.68)	3652	1.38 (1.30-1.47)	<0.001
**Excluding participants with poor self-reported health status at baseline**												
IARC alcohol-related cancers	1847	467	1.00 (0.91-1.10)	453	1.23 (1.12-1.35)	385	1.57 (1.42-1.73)	542	2.09 (1.91-2.30)	1847	1.66 (1.54-1.79)	<0.001
Other cancers of known sites (Non-IARC alcohol-related)	2832	844	1.00 (0.93-1.08)	822	1.24 (1.16-1.32)	578	1.30 (1.20-1.42)	588	1.31 (1.20-1.43)	2832	1.18 (1.09-1.28)	<0.001
All cancers[a]	4402	1261	1.00 (0.94-1.06)	1204	1.22 (1.15-1.29)	886	1.35 (1.27-1.45)	1051	1.56 (1.46-1.67)	4402	1.38 (1.30-1.46)	<0.001
**Excluding participants with self-reported prior chronic disease[b] at baseline**												
IARC alcohol-related cancers	1565	359	1.00 (0.89-1.12)	376	1.27 (1.15-1.40)	333	1.65 (1.48-1.83)	497	2.28 (2.07-2.51)	1565	1.74 (1.60-1.89)	<0.001
Other cancers of known sites (Non-IARC alcohol-related)	2328	645	1.00 (0.92-1.09)	674	1.23 (1.14-1.33)	504	1.34 (1.23-1.46)	505	1.29 (1.17-1.41)	2328	1.17 (1.07-1.27)	<0.001
All cancers[a]	3652	965	1.00 (0.93-1.07)	989	1.22 (1.15-1.30)	772	1.41 (1.31-1.51)	926	1.59 (1.49-1.71)	3652	1.39 (1.31-1.49)	<0.001
**Excluding all of above**												
IARC alcohol-related cancers	1129	270	1.00 (0.88-1.14)	267	1.21 (1.08-1.37)	237	1.56 (1.38-1.77)	355	2.16 (1.92-2.42)	1129	1.70 (1.54-1.88)	<0.001
Other cancers of known sites (Non-IARC alcohol-related)	1728	480	1.00 (0.91-1.10)	501	1.23 (1.13-1.34)	372	1.32 (1.19-1.46)	375	1.29 (1.15-1.43)	1728	1.17 (1.06-1.30)	0.002
All cancers[a]	2653	716	1.00 (0.92-1.08)	714	1.19 (1.11-1.28)	555	1.35 (1.24-1.47)	668	1.54 (1.42-1.68)	2653	1.37 (1.27-1.48)	<0.001

HR, hazard ratio; CI, confidence interval; IARC, International Agency for Research on Cancer.

Cox models are stratified by age-at-risk and study area, and adjusted for education, income, smoking, physical activity, fruit intake, body mass index, and family history of cancer.

Participants with self-reported prior cancer were excluded from all analyses.

[a] All cancers included ill-defined neoplasm and are patient-based.

[b] Chronic diseases included self-reported coronary heart disease, stroke, transient ischaemic attack, diabetes, tuberculosis, chronic hepatitis/liver cirrhosis, rheumatoid arthritis, peptic ulcer, emphysema/bronchitis, gallstone/gallbladder disease, and kidney disease.

Table S15. Population attributable fraction of ever-regular drinking on incidence of common cancers

Cancer site	N	HR (95% CI)	Proportion of ever regular drinkers	PAF[a] (approximate 95% CI), %
Mouth and throat	541	1.48 (1.23-1.77)	0.55	17.7 (10.8-24.6)
Oesophagus	1608	1.60 (1.43-1.78)	0.50	18.8 (15.2-22.3)
Colon and rectum	1527	1.26 (1.13-1.40)	0.51	10.4 (6.0-14.8)
Colon	856	1.21 (1.05-1.39)	0.49	8.5 (2.6-14.3)
Rectum	946	1.30 (1.14-1.49)	0.53	12.3 (6.7-17.9)
Liver	1651	1.23 (1.11-1.37)	0.47	8.9 (4.8-13.0)
Stomach	2221	1.10 (1.00-1.20)	0.45	4.0 (0.2-7.8)
Lung	2741	1.12 (1.04-1.22)	0.50	5.4 (1.9-9.0)
IARC alcohol-related cancers	5403	1.33 (1.26-1.41)	0.50	12.4 (10.2-14.6)
Other cancers of known sites (Non-IARC alcohol-related)	8730	1.10 (1.05-1.15)	0.47	4.2 (2.2-6.1)
All cancers[b]	13342	1.18 (1.13-1.22)	0.48	7.2 (5.7-8.7)

HR, hazard ratio; CI, confidence interval; PAF, population attributable fraction; IARC, International Agency for Research on Cancer.

Cox models are stratified by age-at-risk and study area, and adjusted for education, income, smoking, physical activity, fruit intake, body mass index, and family history of cancer. Participants with self-reported prior cancer were excluded from all analyses. Participants with self-reported prior chronic hepatitis/liver cirrhosis were further excluded from analysis of liver cancer, and participants with self-reported prior tuberculosis, emphysema/bronchitis, or chronic obstructive pulmonary disease were further excluded from analysis of lung cancer.

[a] PAF is calculated as P(HR-1)/HR, where P is the proportion of ever-regular alcohol drinkers (i.e. current and ex-regular drinkers) among those who developed the relevant cancer during follow-up.

[b] All cancers included ill-defined neoplasm and are patient-based.

Table S16. Adjusted HRs for cancer mortality associated with alcohol drinking status, in men

Cancer site	All men N	Abstainers N	Abstainers HR (95% CI)	Ex-regular drinkers N	Ex-regular drinkers HR (95% CI)	Occasional drinkers N	Occasional drinkers HR (95% CI)	Current regular drinkers N	Current regular drinkers HR (95% CI)	*P*[a]
Mouth and throat	200	38	1.00 (0.72-1.40)	24	1.31 (0.87-1.96)	46	0.98 (0.72-1.33)	92	1.76 (1.41-2.20)	0.006
Oesophagus	966	150	1.00 (0.84-1.18)	102	1.38 (1.13-1.67)	297	1.00 (0.88-1.13)	417	1.95 (1.76-2.17)	<0.001
Colon and rectum	499	102	1.00 (0.81-1.23)	77	1.46 (1.16-1.83)	148	1.03 (0.86-1.22)	172	1.16 (0.99-1.35)	0.28
Colon	199	40	1.00 (0.72-1.39)	32	1.71 (1.20-2.43)	59	1.04 (0.79-1.37)	68	1.27 (0.99-1.63)	0.25
Rectum	300	62	1.00 (0.77-1.30)	45	1.31 (0.97-1.76)	89	1.01 (0.81-1.26)	104	1.08 (0.88-1.33)	0.64
Liver	1208	279	1.00 (0.88-1.14)	154	1.27 (1.08-1.49)	368	0.85 (0.77-0.95)	407	1.07 (0.96-1.19)	0.42
Stomach	1227	338	1.00 (0.89-1.12)	141	0.96 (0.81-1.14)	381	0.80 (0.72-0.89)	367	0.89 (0.80-0.99)	0.16
Pancreas	285	61	1.00 (0.77-1.30)	28	0.96 (0.66-1.40)	79	1.01 (0.80-1.27)	117	1.27 (1.05-1.55)	0.15
Lung	1768	442	1.00 (0.91-1.10)	224	1.03 (0.90-1.17)	444	0.77 (0.70-0.85)	658	0.97 (0.89-1.05)	0.60
Gallbladder	102	13	1.00 (0.57-1.76)	14	1.92 (1.13-3.27)	32	1.90 (1.30-2.78)	43	2.05 (1.50-2.80)	0.03
Prostate	131	36	1.00 (0.71-1.42)	27	1.71 (1.16-2.52)	38	1.00 (0.71-1.39)	30	0.83 (0.57-1.19)	0.47
Kidney	37	9	1.00 (0.49-2.05)	6	1.46 (0.65-3.30)	11	0.64 (0.34-1.19)	11	0.83 (0.44-1.56)	0.70
Bladder	91	25	1.00 (0.65-1.53)	16	1.13 (0.69-1.86)	25	0.82 (0.54-1.24)	25	0.73 (0.49-1.11)	0.31
Brain	126	25	1.00 (0.66-1.51)	15	1.23 (0.73-2.05)	46	1.07 (0.78-1.46)	40	1.07 (0.77-1.49)	0.80
Lymphoma	76	18	1.00 (0.62-1.63)	11	1.29 (0.71-2.36)	18	0.76 (0.47-1.24)	29	1.10 (0.75-1.61)	0.77
Multiple myeloma and malignant plasma cell neoplasms	65	19	1.00 (0.62-1.61)	4	0.46 (0.17-1.22)	18	0.74 (0.46-1.20)	24	0.88 (0.58-1.34)	0.70
Leukaemia	145	38	1.00 (0.71-1.40)	15	0.86 (0.51-1.43)	41	0.75 (0.54-1.04)	51	0.95 (0.71-1.27)	0.82
IARC alcohol-related cancers	3044	617	1.00 (0.92-1.09)	401	1.36 (1.23-1.50)	904	0.89 (0.83-0.95)	1122	1.27 (1.19-1.35)	<0.001
Other cancers of known sites (Non-IARC alcohol-related)	4899	1261	1.00 (0.94-1.06)	627	1.03 (0.95-1.12)	1321	0.81 (0.76-0.86)	1690	0.98 (0.93-1.03)	0.56
All cancers[b]	8016	1895	1.00 (0.95-1.05)	1033	1.13 (1.06-1.20)	2250	0.83 (0.79-0.87)	2838	1.07 (1.03-1.11)	0.04

HR, hazard ratio; CI, confidence interval; IARC, International Agency for Research on Cancer.

Cox models are stratified by age-at-risk and study area, and adjusted for education, income, smoking, physical activity, fruit intake, body mass index, and family history of cancer.

Participants with prior self-reported cancers were excluded from all analyses. Participants with prior self-reported chronic hepatitis/cirrhosis were further excluded from analysis of liver cancer, and participants with prior self-reported tuberculosis, emphysema/bronchitis, or chronic obstructive pulmonary disease were further excluded from analysis of lung cancer.

[a] *P* for association comparing current regular drinkers vs. abstainers.

[b] All cancers included ill-defined neoplasm and are patient-based.

Table S17. Adjusted HRs for cancer mortality associated with level of alcohol consumption, in male current regular drinkers

		<140 g/week		140-279 g/week		280-419 g/week		420+ g/week		Per 280 g/week usual intake		
**Cancer site**	**All current regular N**	**N**	**HR (95% CI)**	**N**	**HR (95% CI)**	**N**	**HR (95% CI)**	**N**	**HR (95% CI)**	**N**	**HR (95% CI)**	P_{trend}
Mouth and throat	92	13	1.00 (0.56-1.77)	27	2.27 (1.56-3.32)	17	2.27 (1.41-3.64)	35	3.83 (2.65-5.54)	92	1.68 (1.29-2.19)	<0.001
Oesophagus	417	64	1.00 (0.77-1.30)	90	1.67 (1.36-2.05)	81	2.19 (1.77-2.72)	182	4.21 (3.55-5.00)	417	2.04 (1.81-2.29)	<0.001
Colon and rectum	172	54	1.00 (0.75-1.34)	49	1.18 (0.89-1.55)	32	1.19 (0.84-1.69)	37	1.30 (0.92-1.85)	172	1.38 (1.03-1.86)	0.03
Colon	68	27	1.00 (0.65-1.54)	16	0.81 (0.50-1.32)	15	1.26 (0.75-2.10)	10	0.85 (0.44-1.65)	68	1.11 (0.63-1.93)	0.72
Rectum	104	27	1.00 (0.67-1.50)	33	1.53 (1.09-2.14)	17	1.16 (0.72-1.87)	27	1.68 (1.11-2.54)	104	1.53 (1.08-2.16)	0.02
Liver	407	108	1.00 (0.81-1.23)	97	1.16 (0.95-1.41)	90	1.62 (1.32-1.99)	112	1.93 (1.57-2.36)	407	1.58 (1.34-1.87)	<0.001
Stomach	367	121	1.00 (0.82-1.22)	85	0.83 (0.68-1.03)	83	1.19 (0.96-1.48)	78	1.04 (0.82-1.32)	367	1.14 (0.91-1.43)	0.25
Pancreas	117	37	1.00 (0.70-1.44)	30	0.91 (0.64-1.29)	20	0.90 (0.58-1.41)	30	1.31 (0.89-1.94)	117	1.18 (0.79-1.77)	0.41
Lung	658	210	1.00 (0.86-1.16)	174	1.09 (0.94-1.26)	138	1.41 (1.19-1.67)	136	1.38 (1.15-1.66)	658	1.29 (1.10-1.51)	0.002
Gallbladder	43	15	1.00 (0.56-1.80)	10	0.80 (0.43-1.47)	9	1.13 (0.58-2.18)	9	0.94 (0.46-1.92)	43	0.93 (0.45-1.90)	0.84
Prostate	30	10	1.00 (0.49-2.05)	11	2.08 (1.17-3.69)	4	1.35 (0.50-3.67)	5	1.56 (0.61-4.03)	30	1.22 (0.56-2.68)	0.62
Bladder	25	9	1.00 (0.48-2.10)	7	0.83 (0.41-1.71)	2	0.31 (0.08-1.23)	7	0.88 (0.38-2.08)	25	0.74 (0.28-2.00)	0.56
Brain	40	8	1.00 (0.48-2.07)	14	2.14 (1.27-3.61)	11	2.51 (1.38-4.56)	7	1.57 (0.73-3.38)	40	1.14 (0.58-2.23)	0.70
Lymphoma	29	7	1.00 (0.45-2.23)	7	1.10 (0.53-2.28)	4	0.77 (0.29-2.05)	11	1.83 (0.95-3.52)	29	1.58 (0.86-2.93)	0.14
Myeloma	24	9	1.00 (0.47-2.11)	7	1.29 (0.63-2.66)	3	0.85 (0.27-2.65)	5	1.42 (0.54-3.73)	24	1.10 (0.43-2.78)	0.84
Leukaemia	51	19	1.00 (0.60-1.67)	13	0.99 (0.58-1.69)	10	1.21 (0.65-2.27)	9	1.11 (0.55-2.22)	51	1.41 (0.88-2.24)	0.15
IARC alcohol-related cancers	1122	252	1.00 (0.87-1.14)	270	1.33 (1.18-1.50)	227	1.68 (1.48-1.91)	373	2.48 (2.21-2.78)	1122	1.76 (1.61-1.91)	<0.001
Other cancers of known sites (non-IARC alcohol-related)	1690	552	1.00 (0.91-1.10)	439	1.02 (0.93-1.12)	343	1.24 (1.12-1.38)	356	1.23 (1.10-1.38)	1690	1.20 (1.08-1.32)	<0.001
All cancers[a]	2838	811	1.00 (0.93-1.08)	713	1.11 (1.04-1.20)	576	1.39 (1.28-1.51)	738	1.65 (1.52-1.79)	2838	1.46 (1.36-1.56)	<0.001

HR, hazard ratio; CI, confidence interval; IARC, International Agency for Research on Cancer.

Cox models are stratified by age-at-risk and region, and adjusted for education, income, smoking, physical activity, fruit intake, body mass index, and family history of cancer.

Participants with self-reported prior cancer were excluded from all analyses. Participants with self-reported prior chronic hepatitis/liver cirrhosis were further excluded from analysis of liver cancer, and participants with self-reported prior tuberculosis, emphysema/bronchitis, or chronic obstructive pulmonary disease were further excluded from analysis of lung cancer.

[a] All cancers included ill-defined neoplasm and are patient-based.

Table S18. Reproducibility of self-reported alcohol consumption data in the CKB

	Baseline and quality control survey (mean 17 days apart)		Baseline (2004-08) and 1st resurvey (2008) (mean 2.6 years apart)		
	N	Weighted kappa coefficient	N	Weighted kappa coefficient	Pearson's correlation coefficient
Drinking frequency in the past 12 months	15718	0.79	19786	0.64	--
**Among current regular drinkers in both surveys**					
Drinking days per week	--	--	1952	0.48	--
Beverage type	--	--	1952	0.59	--
Total alcohol intake, g/week	--	--	1952	--	0.58

Table S19. Prevalence of alcohol consumption at baseline (2004-2008), first (2008) and second resurveys (2013-2014), by sex

	Men			Women		
	Baseline	1st Resurvey	2nd Resurvey	Baseline	1st Resurvey	2nd Resurvey
Number of participants	210205	7772	9579	302510	12014	15462
Drinking category, %						
Abstainers	20.4	22.9	34.9	63.6	66.9	81.9
Ex-regular drinkers	8.7	8.3	10.1	0.9	0.8	1.3
Occasional drinkers	37.7	38.9	26.4	33.5	30.5	14.9
Current regular drinkers	33.3	29.9	28.6	2.1	1.9	1.9

Prevalence was based on cross-sectional data reported at the time of each survey.

Table S20. Patterns of alcohol consumption at baseline (2004-2008), first (2008) and second resurveys (2013-2014), among current regular drinkers, by sex

	Men			Women		
	Baseline	1st Resurvey	2nd Resurvey	Baseline	1st Resurvey	2nd Resurvey
Number of participants	69897	2319	2735	6245	226	293
Mean intake, g/week	285.7	278.9	352.0	115.6	139.5	129.9
Consumption level in g/week, %						
<140	35.9	37.5	26.9	77.0	72.1	72.3
140-279	27.1	26.6	27.4	15.9	19.9	17.8
280-419	18.4	17.5	19.6	4.7	3.5	5.5
420+	18.7	18.4	26.1	2.4	4.4	4.4
Types consumed on typical day, %						
Spirits only	69.6	64.8	52.9	61.8	63.3	46.4
Beer only	18.2	22.2	8.2	22.2	23.0	10.6
Rice wine or grape wine only	12.2	13.0	10.9	16	13.7	26.6
Mixed	--	--	27.9	--	--	16.4
Drinking patterns, %						
Daily drinking	62.1	65.0	71.1	45.3	57.1	62.8
Heavy episodic drinking	37.2	36.1	45.5	26.5	31.0	24.6
Drinking outside meals	14.1	11.9	17.6	13.8	5.8	20.8
Flushing after drinking, %	17.9	14.1	15.5	23.6	23.5	14.0

Prevalence and mean were based on cross-sectional data reported at the time of each survey. Participants were allowed to report only one beverage type at baseline and the first resurvey, and up to three beverage types at the second resurvey.

Table S21. Associations of blood pressure and gamma-glutamyl transferase with alcohol consumption among men at baseline

	N	Abstainers	Ex-regular drinkers	Occasional drinkers	Current regular drinkers <140 g/week	140-279 g/week	280-419 g/week	420+ g/week	P_{trend}[a]
Mean SBP, mmHg (SE)	199242	131.6 (0.1)	133.5 (0.1)	130.7 (0.1)	132.0 (0.1)	134.5 (0.1)	136.1 (0.2)	137.2 (0.2)	<.0001
Mean DBP, mmHg (SE)	199242	78.3 (0.1)	79.7 (0.1)	77.9 (0.0)	79.2 (0.1)	80.7 (0.1)	81.9 (0.1)	82.5 (0.1)	<.0001
Mean GGT, IU/L (SE)	8574	29.6 (2.2)	33.5 (3.1)	29.9 (1.9)	43.7 (3.1)	58.7 (3.5)	83.0 (4.2)	118.6 (4.2)	<.0001

SBP, systolic blood pressure; DBP, diastolic blood pressure; GGT, gamma-glutamyl transferase; SE, standard error.
Participants with prior cancer, coronary heart disease, stroke or transient ischaemic attack were excluded.
Mean values are adjusted for age, region, education, income, and smoking. Means values for blood pressure are further adjusted for physical activity and seasonality.
[a] *P* for trend among current regular drinkers.

References

1. European Association for the Study of the Liver, European Organisation for Research and Treatment of Cancer. EASL-EORTC clinical practice guidelines: management of hepatocellular carcinoma. *Journal of hepatology* 2012;**56**: 908-43.

2. Brenner DR, McLaughlin JR, Hung RJ. Previous lung diseases and lung cancer risk: a systematic review and meta-analysis. *PloS one* 2011;**6**: e17479.

3. Zhang Q, Li L, Smith M, et al. Exhaled carbon monoxide and its associations with smoking, indoor household air pollution and chronic respiratory diseases among 512,000 Chinese adults. *Int J Epidemiol* 2013;**42**: 1464-75.

4. Chen ZM, Peto R, Iona A, et al. Emerging tobacco-related cancer risks in China: A nationwide, prospective study of 0.5 million adults. *Cancer* 2015;**121 Suppl 17**: 3097-106.

5. Chen Z, Peto R, Zhou M, et al. Contrasting male and female trends in tobacco-attributed mortality in China: evidence from successive nationwide prospective cohort studies. *Lancet* 2015;**386**: 1447-56.

6. World Cancer Research Fund/American Institute for Cancer Research, Continuous Update Project Expert Report 2018. Alcoholic drinks and the risk of cancer, 2018.

7. Millwood IY, Li L, Smith M, et al. Alcohol consumption in 0.5 million people from 10 diverse regions of China: prevalence, patterns and socio-demographic and health-related correlates. *International journal of epidemiology* 2013;**42**: 816-27.

8. Im PK, Millwood IY, Guo Y, et al. Patterns and trends of alcohol consumption in rural and urban areas of China: findings from the China Kadoorie Biobank. *BMC public health* 2019;**19**: 217.

9. International Agency for Research on Cancer, IARC monographs on the evaluation of carcinogenic risks to humans 100E Personal habits and indoor combustions. International Agency for Research on Cancer, 2012.

10. Easton DF, Peto J, Babiker AG. Floating absolute risk: an alternative to relative risk in survival and case-control analysis avoiding an arbitrary reference group. *Statistics in medicine* 1991;**10**: 1025-35.

11. Clarke R, Shipley M, Lewington S, et al. Underestimation of risk associations due to regression dilution in long-term follow-up of prospective studies. *Am J Epidemiol* 1999;**150**: 341-53.

12. MacMahon S, Peto R, Cutler J, et al. Blood pressure, stroke, and coronary heart disease. Part 1, Prolonged differences in blood pressure: prospective observational studies corrected for the regression dilution bias. *Lancet* 1990;**335**: 765-74.

13. Rosner B, Willett WC, Spiegelman D. Correction of logistic regression relative risk estimates and confidence intervals for systematic within-person measurement error. *Statistics in medicine* 1989;**8**: 1051-73.

14. Cochrane J, Chen H, Conigrave KM, et al. Alcohol use in China. *Alcohol Alcohol* 2003;**38**: 537-42.

15. World Health Organization, International Guide for Monitoring Alcohol Consumption and Related Harm. World Health Organization, 2000.
